# Supramolecular Polymer Intertwined Free-Standing Bifunctional Membrane Catalysts for All-Temperature Flexible Zn–Air Batteries

**DOI:** 10.1007/s40820-022-00927-0

**Published:** 2022-09-17

**Authors:** Nayantara K. Wagh, Sambhaji S. Shinde, Chi Ho Lee, Sung-Hae Kim, Dong-Hyung Kim, Han-Don Um, Sang Uck Lee, Jung-Ho Lee

**Affiliations:** 1 Department of Materials Science and Chemical Engineering, Hanyang University, Ansan, Republic of Korea; 2 Department of Applied Chemistry, Center for Bionano Intelligence Education and Research, Hanyang University, Ansan, Republic of Korea; 3 Department of Chemical Engineering, Kangwon National University, Chuncheon, Gangwon 24341 Republic of Korea

**Keywords:** Flexible free-standing membrane electrocatalysts, Supramolecular polymer, Alkaline and flexible solid-state Zn–air batteries, All-temperature operations, High capacity and energy density

## Abstract

Rational construction of flexible free-standing electrocatalysts featuring long-lasting durability, high efficiency, and wide temperature tolerance under harsh practical operations are fundamentally significant for commercial zinc–air batteries. Here, 3D flexible free-standing bifunctional membrane electrocatalysts composed of covalently cross-linked supramolecular polymer networks with nitrogen-deficient carbon nitride nanotubes are fabricated (referred to as PEMAC@NDCN) by a facile self-templated approach. PEMAC@NDCN demonstrates the lowest reversible oxygen bifunctional activity of 0.61 V with exceptional long-lasting durability, which outperforms those of commercial Pt/C and RuO_2_. Theoretical calculations and control experiments reveal the boosted electron transfer, electrolyte mass/ion transports, and abundant active surface site preferences. Moreover, the constructed alkaline Zn–air battery with PEMAC@NDCN air–cathode reveals superb power density, capacity, and discharge–charge cycling stability (over 2160 cycles) compared to the reference Pt/C + RuO_2_. Solid-state Zn–air batteries enable a high power density of 211 mW cm^−2^, energy density of 1056 Wh kg^−1^, stable charge–discharge cycling of 2580 cycles for 50 mA cm^−2^, and wide temperature tolerance from − 40 to 70 °C with retention of 86% capacity compared to room-temperature counterparts, illustrating prospects over harsh operations.

**Supplementary Information:**

The online version contains supplementary material available at 10.1007/s40820-022-00927-0.

## Highlights

- Rational design of 3D flexible free-standing membrane catalysts with protonation between supramolecular polymers and nitrogen-deficient carbon nitride nanotubes frameworks via facile bottom-up self-conversion approach.
- PEMAC@NDCN demonstrates the lowest reversible oxygen bifunctional activity of 0.61 V with exceptional long-lasting durability, surpassing the commercial and reported champion oxygen catalysts.
- Engineering catalytically active cathode materials enabled all-temperature flexible Zn–air batteries with high electrochemical and mechanical performances (temperature of − 40 to 70 °C) under harsh operations.

**Supplementary Information:**

The online version contains supplementary material available at 10.1007/s40820-022-00927-0.

## Graphical abstract

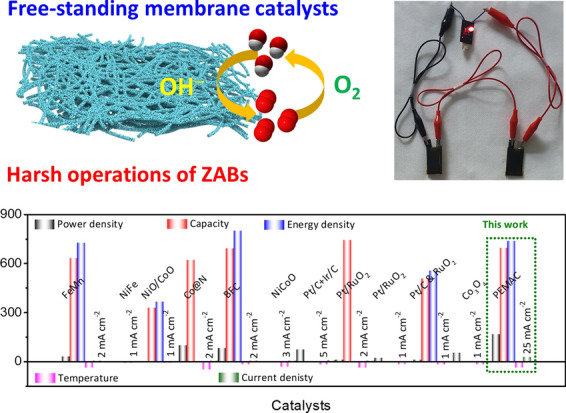

## Introduction

The global increment of electrical transportation and wearable/portable electronics technology urgently demands high-energy flexible rechargeable batteries that are safe and operate over wide temperatures and harsh conditions [1–5]. Commercial Li-ion battery operations are strongly restricted to the temperature range of − 20 to + 15 °C with severe reduction in energy and power densities [6]. Among the aqueous/non-aqueous metal–air batteries (alkali metals: Li, Na, and K), alkaline earth metals (Mg) and transition or post-transition metals (Fe, Zn, Al, Sn), Zn–air batteries (ZABs) with earth-abundant Zn resources, high theoretical energy density (1086 Wh kg^−1^), cost-effectiveness, and intrinsic safety are the most competitive energy storage systems to satisfy the growing practical requirements [7–10].

However, the large overpotentials, inferior rate-capability, and unsustainable electrochemical stability during discharge–charge states over a wide temperature range due to the decelerated reaction kinetics of air cathodes and reduced ion conductivity of electrolytes remain the most critical challenges for practical applications [11–14]. Pt-, Ir/Ru-based materials are the present benchmark catalysts for oxygen reduction/evolution reactions (ORR/OER). The poor economic viability, time-drifting catalytic efficiency, scarcity, and singular catalytic characters (Pt/C for ORR or HER, Ir/Ru for OER), however, limit the scalable deployment of ZABs [15–18]. Extensive efforts have been directed to enlarge the operational efficiencies of bifunctional catalysts to boost the electrochemical kinetics of flexible ZABs. Most typical methods are transition metal-based materials (phosphides, sulfides, oxides, nitrides, hydroxides, oxy-hydroxides) and composites with heteroatoms-doped carbon [M-(P, S, O, N, OH, OOH)-C], transition metal–nitrogen-doped carbon (M–N–C), and metal-free carbon-based frameworks [1–27]. However, these approaches severely deteriorate the capacity and cycle life of ZABs due to the densely packed aggregation, inferior intrinsic electrochemical activities or stability, reactants inaccessibility, and complex design processes for harsh operating conditions. Moreover, the ZABs operations for wide temperatures are still in the first phase due to limited temperature-resistant catalysts and electrolytes resources. Therefore, insights for the rational design of competitive oxygen bifunctional catalysts to offset the wide temperature performance loss are highly desirable for exceptional operations of ZABs.

Recently, most reported electrodes are integrated with polymer binders and then dispersed on the conductive substrates. The loading of binders will inevitably lead to the increase in oxygen transport resistance with deterioration for active surface sites, thus causing large overpotentials, reduction in ZABs capacities and long-term operations [5–7]. Therefore, constructing self-standing, highly efficient oxygen bifunctional membrane electrodes with intrinsic bendability is one of the alternative strategies. The nanostructured supramolecular polymers have attracted significant attention as new emerging energy materials owing to the merits of large surface area, ease of functionalization, high compositional tunability, and facile synthesis process. The structural uncertainty, poor crystallinity, inferior electrical conductivity for electron transport, interfacial features to accommodate large deformation, and poor temperature tolerance of supramolecular polymers are the critical issues for the fabrication of competent oxygen bifunctional catalysts with capability for extremely harsh mechanical, environmental, and electrochemical operations [4, 5, 10]. These demerits of supramolecular polymers can be diminished by cross-linking of deicing mediators such as metal-free or metallic frameworks into the polymers. The self-assembly of small molecules with reversible covalent/non-covalent interactions is not considered for global roadmaps due to poor processability despite a large degree of freedom for environmental compatibility and resources. Primarily, defect engineering, enhancing the number of reactive sites, and their intrinsic activity is the fundamental principles of developing efficient catalysts [28]. Graphitic carbon nitride (g-C_3_N_4_) possesses highly conjugated electronic structures and high N content with a large number of lone-pair electrons, thereby promoting abundant anchoring sites [26]. However, irreversible reactions of graphitic nitrogen facilitate the structural disorder causing severe irreversible capacity loss. Therefore, designing one-dimensional nitrogen-deficient carbon nitride nanotubes (1D-NDCN) frameworks with abundant active edges is the paramount approach to determine an exceptional electrochemical activity and durability. Notably, the versatile molecular building blocks for 1D-NDCN offer optimal structural and electronic properties over atomic precision and favorable HOMO/LUMO orbital distributions [29]. It accelerates the adsorption/desorption behavior of oxygen intermediates at the carbon atoms, which results in superior ORR and OER activities. However, the conjugated π-electron structures restrict the efficient electron and mass transfer [30–32]. Electronic coupling of supramolecular polymers with 1D-NDCN can overcome the above issues due to intermolecular charge transfer between NDCN and surface adsorbates. A polymeric carbon protects the active interfacial sites, which illustrates superior durability even for harsh reactions. Therefore, the practical design strategy for fabricating free-standing active materials and cell structures with remarkable electrochemical performances and environmental adaptability is vital for ZABs.

Herein, we report that all-temperature flexible ZABs with high electrochemical and mechanical performances (power density of 211 mW cm^−2^, energy density of 1056 Wh kg^−1^, cycle life 2580 cycles for 50 mA cm^−2^, temperature of − 40 to 70 °C) enabled under harsh operational conditions through engineering catalytic active cathode materials. The three-dimensional (3D) flexible free-standing membrane catalysts with protonation between supramolecular polymers [poly(ethylene-*alt*-maleic acid)] and nitrogen-deficient carbon nitride nanotubes (PEMAC@NDCN) frameworks have been prepared via facile two-step bottom-up self-conversion approach. The flexible electrodes have a large surface area of ~ 756 m^2^ g^−1^ which enables a high density of stable and high-efficiency ORR/OER reactive sites as well as favorable mass and charge transfer kinetics. The fabricated PEMAC@NDCN catalyst delivered superb ORR (half-wave potential of 0.87 V) and OER (1.48 V @ 10 mA cm^−2^) bifunctional activity with the lowest reversible overpotential of 0.61 V and outstanding long-term robustness, surpassing those of the commercial Pt/C and RuO_2_ and recently reported champion oxygen catalysts in alkaline suspension. Density functional theory (DFT) calculations reveal the modulation in electronic structures over long-range interactions and partial delocalization of electrons with increased Lewis basicity of NDCN, which augments active oxygen sites and mass transfer. Furthermore, the alkaline ZABs with PEMAC@NDCN membrane displayed high specific capacity and notable cycling life operations (2160 cycles for 20 mA cm^−2^) compared to those of Pt/C + RuO_2_, which illustrates the feasibility of commercial applications.

## Experimental Methods

### Synthesis of Bulk Carbon Nitride

Bulk carbon nitride (Bulk CN) was fabricated by a modified one-step thermal condensation reactions process of melamine. Melamine (1 g) was dispersed in the mixture of ethanol and DI water (1:1, v/v) for 20 min, then concentrated by a rotary evaporator to remove solvents (Product A). The obtained product A was heat treated for 550 °C for 6 h in the ambient air (heating rate 5 °C min^−1^) to obtain bulk CN.

### Synthesis of Porous Nitrogen-deficient Carbon Nitride (NDCN) Nanotubes

The melamine source product A (1 g) was dissolved in the ethylene glycol (20 mL) under continuous stirring over 30 min, then followed the insertion of HNO_3_ (0.2 M, 60 mL) for the precipitation reaction for 1 h for 25 °C. The resulting precipitates were washed chronologically for ethanol and water and vacuum dried at 60 °C. The whitish powder was reacted with ammonium thiosulfate (0.1 M) under stirring at 25 °C for 60 min, then collected by centrifugation and several cleanings by deionized water until complete removal of sulfate ions or byproducts and vacuum dried at 80 °C for 12 h (Product B). The product B was further heated to 400 °C for 2 h under N_2_ atmosphere (heating rate 3 °C min^−1^) to achieve porous nanotubes of NDCN.

### Fabrication of Free-standing PEMAC@NDCN Membrane Catalysts

Flexible free-standing metal-free catalysts were fabricated by using the solution casting process as follows: The stoichiometric amount of poly(ethylene-*alt*-maleic anhydride) was dispersed to a clean reaction vessel followed by insertion of acetone and water (1:1, v/v) solution. The resulting suspension was stirred for 60 min to acquire a homogenous solution. Next, 20 mg guanosine and NDCN (200 mg) were dissolved by gentle sonication for 1 h in a cold bath to obtain a uniform suspension of supramolecular gel. Note the low-power and high-frequency sonication bath was utilized (Elmasonics, 40 kHz). After that, the reaction suspensions were transferred to the clean glass vessels. Composite membranes were achieved after the comprehensive removal of all the solvents and then mechanically peeled off under water clotting. Further, the membranes were treated with the mixture of acetone and water under sonication for 2 h for hydrolysis of poly(ethylene-*alt*-maleic anhydride) to the polyethylene-*alt*-maleic acid to obtain the final product as PEMAC@NDCN. The polymer contents were utilized in the range of 0–10%. For comparison, polyvinyl alcohol (PVA) and polyacrylic acid (PAA) derived membrane catalysts were also prepared and named PVA@NDCN and PAA@NDCN using a similar process. Other experimental details are included in supplementary Note S1.

## Results and Discussions

### Catalysts Design and Characterizations

**Fig. 1:**
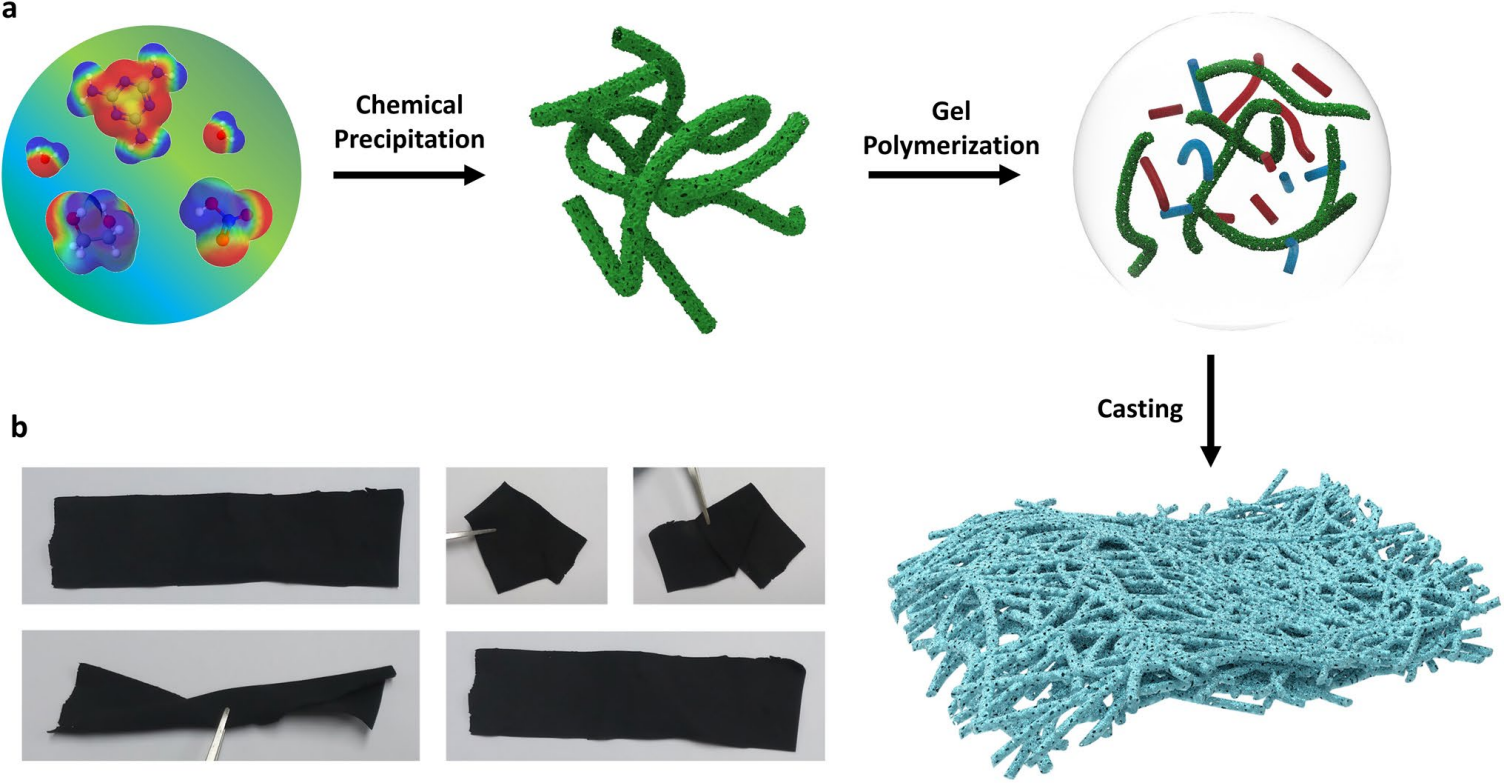
Design illustration for free-standing metal-free catalysts. **a** Schematic process of supramolecular polymer-incorporated nitrogen-deficient carbon–nitrogen frameworks (PEMAC@NDCN) free-standing membrane catalysts. Note the red and blue shapes define the guanosine and PEMAC molecules. **b** The snapshots of flexible free-standing PEMAC@NDCN membrane

Figure 1 presents the fabrication scheme of highly efficient 3D flexible free-standing PEMAC@NDCN membrane bifunctional catalysts via a facile self-templated conversion approach. First, the one-dimensional (1D) porous nitrogen-deficient carbon nitride nanotubes (NDCN) frameworks were prepared by the chemical precipitation of melamine with ethylene glycol, nitric acid, and ammonium thiosulfate with a low-temperature thermo-denitridation process (400 °C). After that, the poly(ethylene-*alt*-maleic anhydride), guanosine, and 1D NDCN frameworks were chemically treated to form the nanotubular structures of supramolecular gels. Then, the gels were interlaced, evaporated the solvents, and mechanically peeled off under water clotting. Finally, 3D flexible PEMAC@NDCN membrane catalysts were obtained through the hydrolysis process to form the cross-linking of polyethylene-*alt*-maleic acid. For comparison, a series of membrane catalysts was also designed. Detailed fabrication processes are presented in the Supporting Information. The porous frameworks of membranes illustrate the abundant electrolyte/electrode interfaces with reducing ion diffusion pathways and a large amount of charge storage, which enables fast electrochemical kinetics and superb driving capability.

**Fig. 2:**
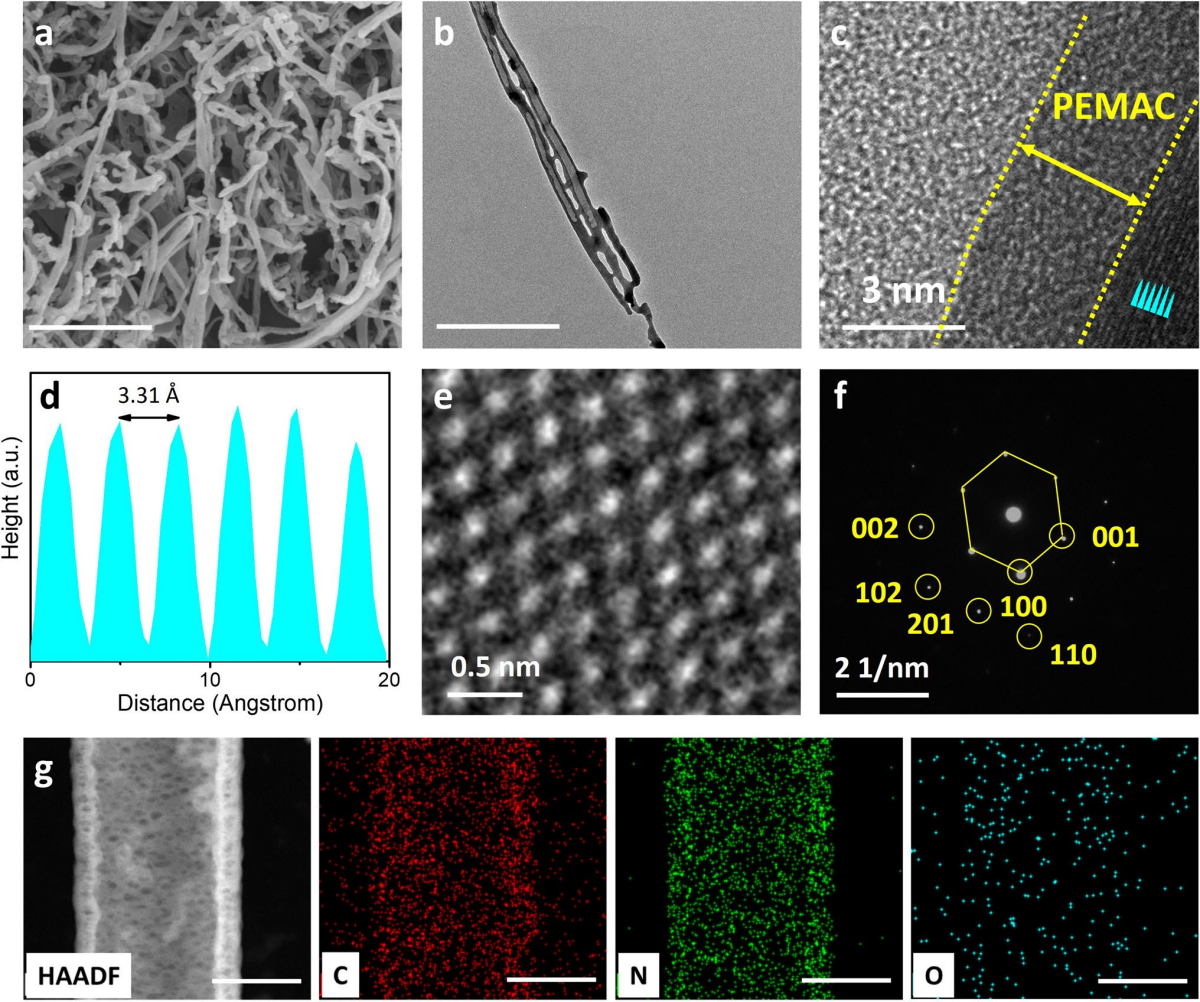
Structural characterizations of PEMAC@NDCN. **a** SEM image and **b** STEM image of PEMAC@NDCN free-standing catalysts. **c** High-resolution STEM image. **d** Topographic interplanar spacing profiles of stated cyan region in (**c**). **e** Enlarged HRTEM image defining hexagonal crystal structure. **f** SAED pattern along with different crystal orientations. **g** HAADF-STEM image with elemental maps of C, N, and O in the PEMAC@NDCN. Scale bars, **a** 500 nm, **b** 300 nm, and **g** 50

**Fig. 3:**
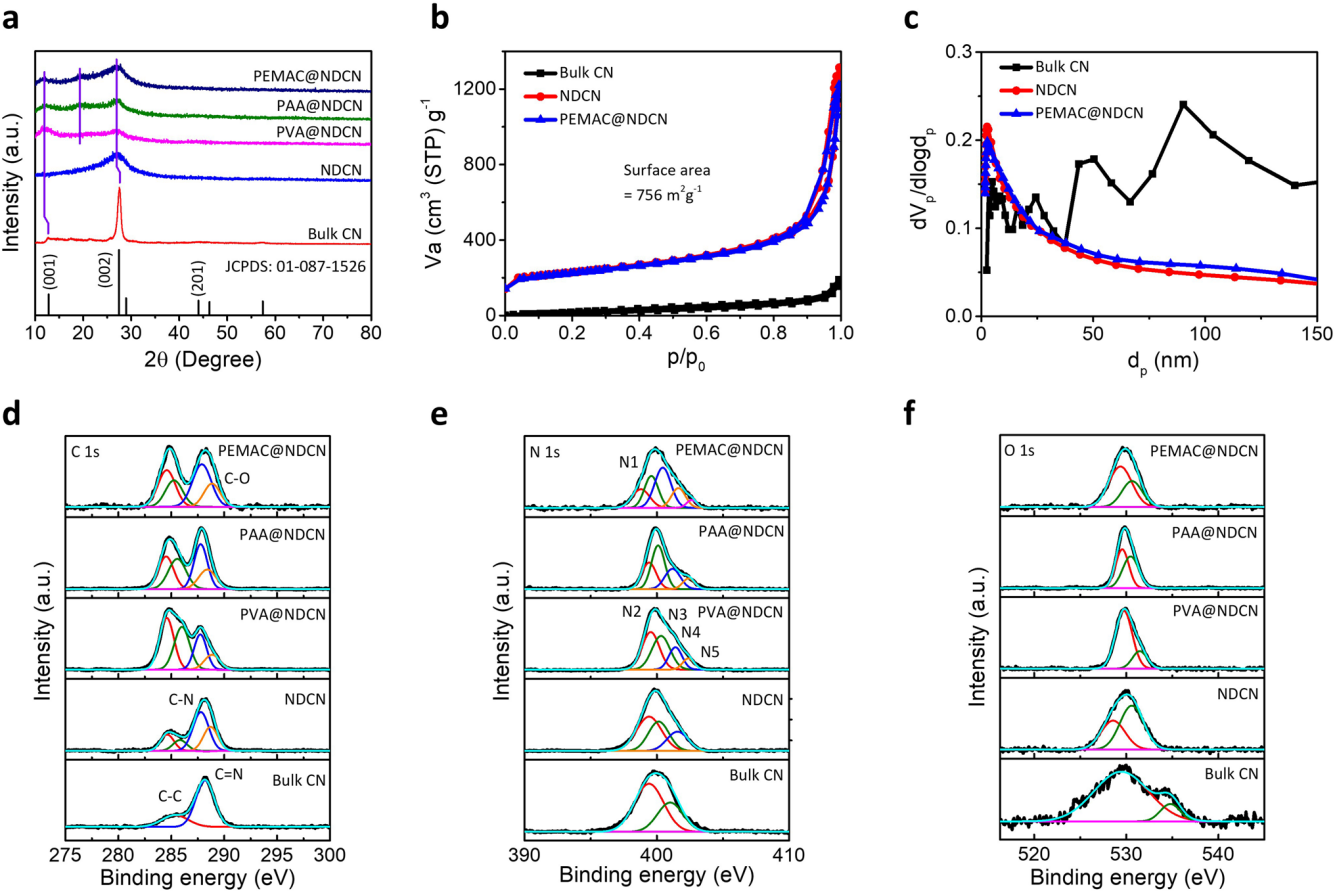
Spectroscopic characterizations of free-standing metal-free catalysts. **a** X-ray diffraction patterns for bulk CN, NDCN, PVA@NDCN, PAA@NDCN, and PEMAC@NDCN catalysts. **b** N_2_ sorption isotherms of PEMAC@NDCN compared to bulk CN and NDCN. **c** Pore distribution statistics of PEMAC@NDCN compared to bulk CN and NDCN. XPS spectra of **d** C 1*s*, **e** N 1*s*, and **f** O 1*s* for the designed PEMAC@NDCN compared to those of other polymers PVA@NDCN, PAA@NDCN, and NDCN, bulk CN

The scanning electron microscopy (SEM, Fig. 2a) and transmission electron microscopy (TEM, Fig. 2b) images clarify the PEMAC@NDCN membrane catalysts exhibit intertwined hierarchical porous nanotubular 3D network structure with uniform distributions of supramolecular polymer over the NDCN surface (diameter ranged in 40–100 nm). The well-distribution of nano-pores (4–5 nm in diameter) promotes the active site exposure, electrolyte infiltration, ion/electron transport pathways, and enlarges the active surface area. High-resolution TEM images (Fig. 2c, d) reveal the formation of uniform distribution of amorphous supramolecular polymer (~ 3 nm in thickness) interfaced on the NDCN frameworks, with the lattice spacing of 0.33 nm corresponding to the (002) crystal plane of carbon [4, 16, 33]. Aberration-corrected TEM image (Fig. 2e) and selected area electron diffraction (SAED, Fig. 2f) pattern confirms the atomically confined hexagonal crystal structure of NDCN skeleton with (002) and (001) reflection as consistent with XRD results (Fig. 3a). High-angle annular dark-field (HAADF-STEM) characterization and elemental maps reveal the uniform distributions of C, N, and O elements throughout the nanotube due to covalent coordination and intense confinement effects of derived open frameworks.

XRD patterns of PEMAC@NDCN (Fig. 3a) display two characteristic broad peaks centered at 11.9° and 26.7°, consistent with the (001) and (002) crystal planes of hexagonal graphitic carbon nitride with shifting towards low diffraction angle compared to those of bulk CN (12.7° and 27.5°, JCPDS No. 01-087-1526). Furthermore, the additional prominent peak at 19.5° was observed for PEMAC@NDCN, PAA@NDCN, and PVA@NDCN, which is the characteristic signal for coaxially grown supramolecular polymeric interfacial chains due to the reduced intrinsic van der Waals forces NDCN [34–36]. This feature illustrates the successful formation of supramolecular polymers over NDCN associated with stacked conjugated aromatic structures (Fig. S1), consistent with TEM and SAED results. The impact of polymer activation displays a smaller diffraction intensity revealing increased structural distortions compared to bulk CN, thereby facilitating local electronic distributions [37]. The nitrogen sorption isotherms and pore distribution characteristics of PEMAC@NDCN and bulk CN are demonstrated in Fig. 3b, c. Well-distinct hysteresis loops reflect the presence of abundant micropores and mesopores. The PEMAC@NDCN determines a significant increase in surface area and pore volume of 756 m^2^ g^−1^ and 0.71 cm^3^ g^−1^, 13.5 and 7.1 times that of bulk CN (56 m^2^ g^−1^, 0.1 cm^3^ g^−1^) and lower than NDCN (779 m^2^ g^−1^). PEMAC@NDCN possesses more dominant micropores/mesopores of ~ 2.7 nm in contrast to the bulk CN, which ensures the efficient accessibility of active sites and mass/charge transfer reactions [37].

X-ray photoelectron spectroscopy (XPS) measurements are performed to detect the chemical bindings, stoichiometric compositions, and electronic structures of C, N, and O atoms in the PEMAC@NDCN free-standing catalysts. The XPS survey spectra reveal the presence of characteristics C (58.9 at%), N (33.24 at%), O (7.86 at%) elemental core-states, which is consistent with EDS analysis (Fig. S2 and Table S1). High-resolution C 1*s* spectra of PEMAC@NDCN (Fig. 3d) confirms the four peaks of C–C (*sp*^2^, 284.6 eV), C–C (*sp*^3^, 285.2 eV), C–N/C=N/C–O (287.9 eV), and O–C=O/O=C–OH (288.9 eV) [11, 32, 38–40]. The strong *π*–*π* interactions among NDCN and polymer species anchor the critical role in forming the interlayered structures [30]. N 1*s* spectra (Figs. 3e, S3, and Table S2) demonstrates the five characteristics core-states of pyridinic N (398.8 eV), pyrrolic N (399.6 eV), graphitic N (400.4 eV), quaternary N (401.6 eV), and oxidized N (402.6 eV) [7, 16, 26, 27, 41, 42]. The pyridinic N transforms the local electronic structure of adjacent carbon (positively charged) stimulating the construction of operational active sites and graphitic N enables the four-electron transfer oxygen reaction kinetics. Furthermore, it also coordinates the *sp*^2^-bonded N with heterocyclic C–N=C and bridging of N(–C)_3_ [43]. The presence of active pyridinic N and the highest total content of graphitic and pyridinic N after activation of PEMAC compared other polymers, NDCN, and bulk CN, in which PEMAC contributes the fixation of N species in the NDCN frameworks during polymerization and hydrolysis processes, thereby ensuring the favorable electronic charge delocalization mechanism. The O 1*s* spectra (Fig. 3f) verify the characteristics of oxygen functional groups of C–O (529.7 eV), and C=O/O–H (530.9 eV) [35, 44]. Total content of oxygen increases in the polymeric catalysts compared to the catalysts without polymer activations as consistent with EDS elemental results (Table S1) and the presence of negatively charged polymeric bindings in the FTIR spectra (Fig. S1), which illustrates the anchoring of polymer chain with oxygen-related bindings. Furthermore, it manifests the chemical bindings and electronic interactions at the interfaces between polymers and NDCN, inducing the redistribution of interfacial charges [45], which promotes the superior charge/ion transfers for electrochemical ORR/OER.

**Fig. 4:**
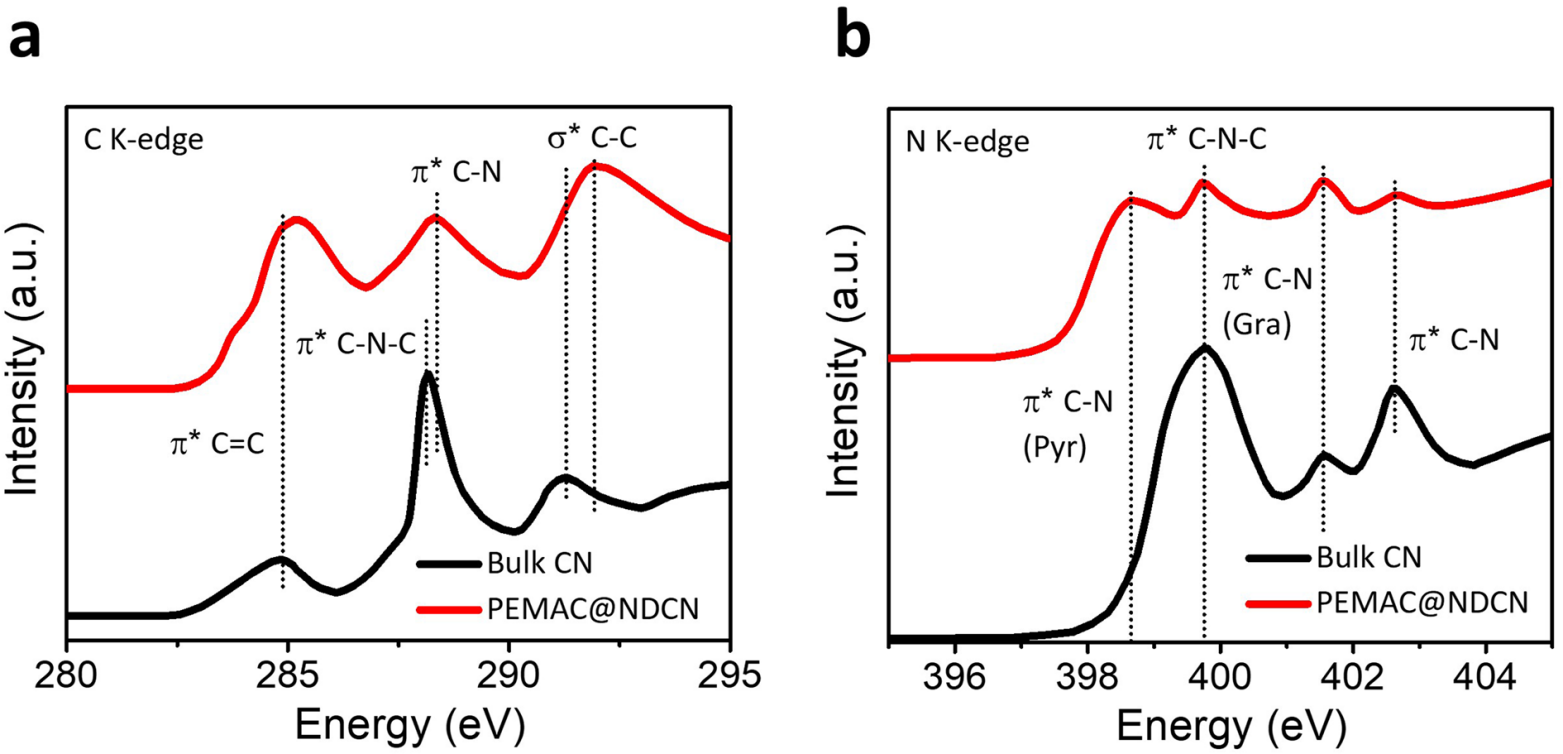
X-ray absorption spectroscopic characterizations. XANES spectra of **a** C K-edge and **b** N K-edge for the PEMAC@NDCN and bulk CN catalysts

As presented in Fig. 4a, C K-edge spectra of PEMAC@NDCN show the main resonances of π* C=C (285.2 eV) and π* C–N (288.5 eV), while the bulk CN displays resonance of π* C–N–C (288.1 eV) [39, 46]. The peak shifting for high-energies of PEMAC@NDCN confirms the dipole transitions of core electrons from C 1*s* to the π* C=C anti-bonding orbitals due to the interfacing for supramolecular polymer chains and withdrawing of N species from bulk CN. N K-edge spectra (Fig. 4b) exhibit the three main resonances of pyridinic π* C–N (398.7 eV), π* C–N–C (399.7 eV), graphitic π* C–N (401.5), and π* C–N (402.6) or N–3C coordination of tri-s-triazine structures [3, 47]. Besides pyridinic π* C–N, all coordinating N resonance has been weakened in PEMAC@NDCN compared to bulk CN, which illustrates the alteration in coordination surroundings and electronic structures for the electrochemically active sites.

### Electrochemical Performance

**Fig. 5:**
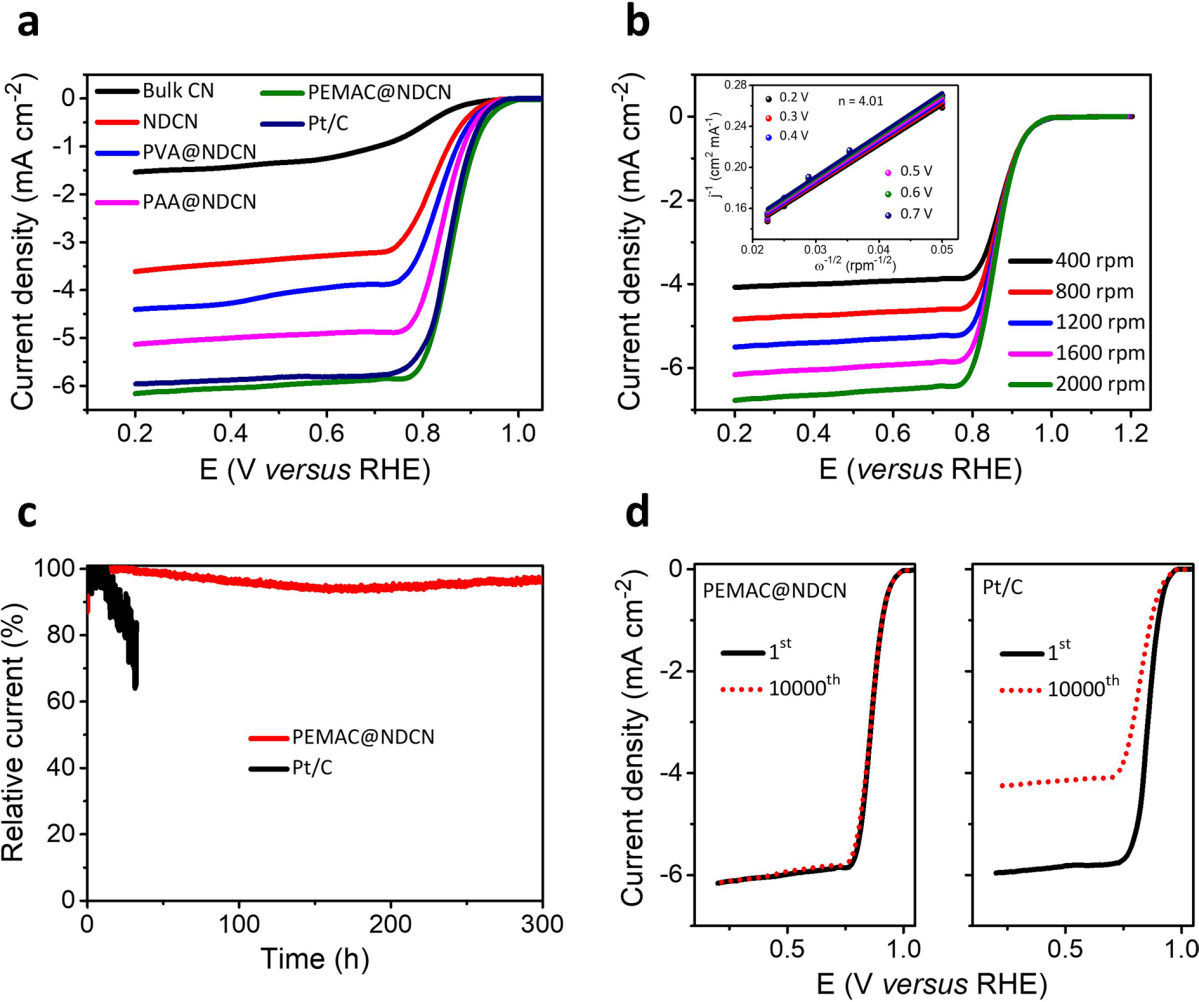
Electrochemical ORR performances for polymer-incorporated metal-free catalysts. **a** LSV profiles for the PEMAC@NDCN compared to those of commercial Pt/C, and other PVA@NDCN, PAA@NDCN, NDCN, bulk CN catalysts. **b** RDE polarizations of PEMAC@NDCN over 400–2000 rpm sweeping rates. Inset displays the characteristics K-L plots for electrochemical kinetics in the range of 0.2–0.7 V potentials of PEMAC@NDCN. **c** Chronopotentiometric polarizations obtained for PEMAC@NDCN compared to those of commercial Pt/C for the respective half-wave potential. **d** Accelerated ORR robustness cyclic operations (initial and after 10,000th) for PEMAC@NDCN compared to those of commercial Pt/C. (Conditions: 0.1 M KOH, RHE scale, RDE: 1600 rpm, current standardized with a geometrical surface area of GCs)

The oxygen bifunctional catalytic performances of designed catalysts are evaluated using a three-electrode system under alkaline environments (O_2_-saturated KOH, 0.1 M). Linear sweep voltammetry (LSV) measurements determine that the PEMAC@NDCN catalyst reveals exceptional ORR performance with a half-wave potential (*E*_1/2_) of 0.87 V and limiting current density of 6.15 mA cm^−2^ compared to those of commercial Pt/C (0.84 V and 5.94 mA cm^−2^) and other champion catalysts (Fig. 5a, S4, and Table S4). Notably, NDCN and bulk CN controls display inferior performance without supramolecular polymer activation, elucidating the significant interfacial characters and limited numbers of competent polymeric active bindings. This feature illustrates that the supramolecular polymers interfaced 3D frameworks guarantee efficient active sites for oxygen transport and rapid kinetics. The PEMAC@NDCN performs the lowest electrochemical impedance compared to those of other prepared catalysts, which indicates the faster charge and mass transport capacities and accelerated ORR reaction kinetics (Fig. S5). To determine the reaction kinetics and electrons transferred number (*n*) of PEMAC@NDCN catalysts during ORR process, the Koutecky–Levich (K–L) profiles are collected for various measured potentials from LSVs performed with different sweeping rates of 400–2000 rpm (Fig. 5b). Linear K–L plots reveal the first-order reaction kinetics for transferred electrons and dissolved O_2_ molecules [2, 7, 48]. The calculated electron transferred number of PEMAC@NDCN is ~ 4.01 (Pt/C of ~ 3.96), implying the selective 4*e*^−^ transfer pathway for ORR (Inset in Figs. 5b and S6). Furthermore, the rotating ring disk electrode (RRDE) measurements are also performed to determine the peroxide yield (HO_2_^−^) and electrons transferred numbers [49]. PEMAC@NDCN has the lowest peroxide yield of ~ 2.5% and transferred electron numbers (3.98–4) in the potential range of 0.2–0.8 V, which is superior to the commercial Pt/C (~ 4.1% and 3.91–3.97). This confirms the high-efficiency 4*e*^−^ reaction process (Fig. S7). *I-t* chronoamperometric profiles (Fig. 5c) of PEMAC@NDCN demonstrate the sustained durability for 300 h with retention of the current density of ~ 97%, surpassing those of commercial Pt/C (32 h, ~ 61%). As shown in Fig. 5d, PEMAC@NDCN reveals superb long-term stability without any obvious decay of *E*_1/2_ even after 10,000 ADT accelerated durability test cycles compared to those of Pt/C (35 mV decay). Furthermore, PEMAC@NDCN shows remarkable tolerance for methanol and CO compared to Pt/C (Fig. S8).

**Fig. 6:**
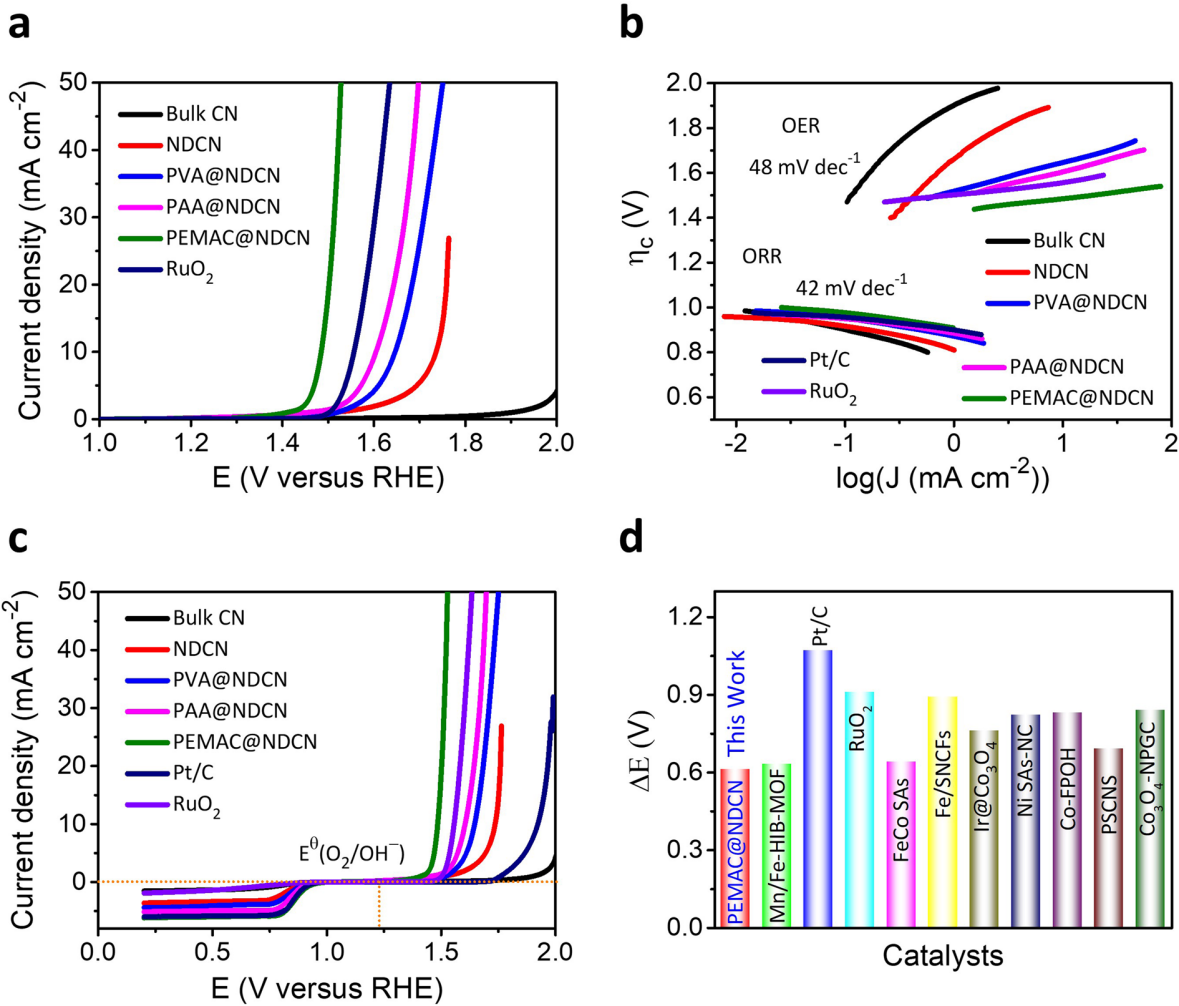
Electrochemical OER performances for visualization as bifunctional metal-free catalysts. **a** LSV profiles for the PEMAC@NDCN compared to those of commercial RuO_2_, and prepared PVA@NDCN, PAA@NDCN, NDCN, bulk CN catalysts. **b** Tafel plots for electrochemical kinetics during ORR and OER for PEMAC@NDCN, PVA@NDCN, PAA@NDCN, NDCN, and bulk CN compared those of commercial reference Pt/C and RuO_2_ materials. **c** Overall bifunctional polarizations of designed polymeric catalysts compared to those of commercial reference Pt/C and RuO_2_ materials. **d** Comparison of oxygen bifunctional reversibility (Δ*E*) of PEMAC@NDCN with reported champion materials (for details see Supplementary Table S4). (Conditions: 0.1 M KOH, RHE scale, RDE: 1600 rpm, current standardized with a geometrical surface area of GCs)

Furthermore, the OER performances are also assessed to validate the bifunctional reactions in alkaline KOH (0.1 M). The PEMAC@NDCN displays a significantly low overpotential of 250 mV for 10 mA cm^−2^ compared to reference RuO_2_ (340 mV) and recently reported superior bifunctional catalysts (Fig. 6a and Table S4). This demonstrates polymer activation is significantly responsible for prominent OER activity relative to NDCN, bulk CN, and RuO_2_ control materials. Moreover, the PEMAC@NDCN reveals the smallest Tafel slopes of 42 mV dec^−1^ (ORR) and 48 mV dec^−1^ (OER) superior to the other PVA@NDCN, PAA@NDCN, and reference materials (Pt/C and RuO_2_), which confirms the most promising ORR and OER kinetics (Fig. 6b). Additionally, the low charge transfer resistance of PEMAC@NDCN catalysts also suggests the superb kinetics during OER process (Fig. S9). The chronoamperometric (*I-t*) measurements and ADT prolongs the retention of the current density of ~ 96% after 300 h and 10,000 ADT cycles with minimal loss in overpotential, illustrating exceptional catalytic durability (Fig. S10). As a reversible O_2_ bifunctional catalysts, PEMAC@NDCN reveals the lowest overall overpotential (Δ*E* = *E*_*j*=10_ − *E*_1/2_) of 0.61 V, which outperforms the reference Pt/C + RuO_2_ and most reported champion bifunctional materials (Figs. 6c, d, and Table S4), illustrating the promising free-standing cathodes for ZABs under harsh operational conditions [1–5, 7–29, 35–49].

### DFT Mechanistic Insights

**Fig. 7:**
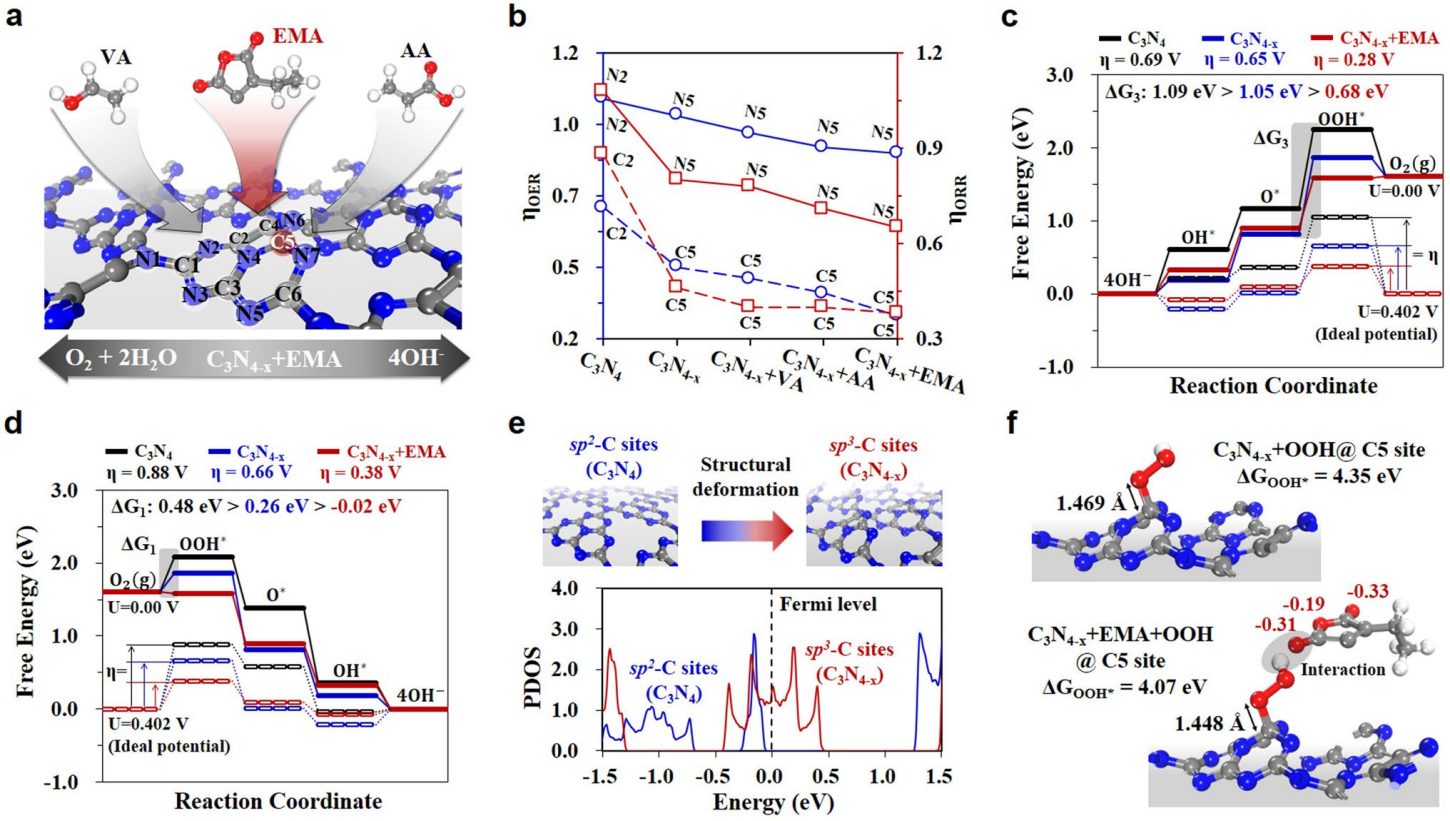
Theoretical mechanistic evaluations. **a** OER/ORR bifunctional polymer-assisted NDCN structures using polymer unit monomer, where VA, AA and EMA represent monomers of poly(vinyl alcohol), poly(acrylic acid) and poly(ethylene-*alt*-maleic acid), respectively, and insets indicate active sites with element symbol and Arabic number. **b** OER and ORR catalytic activity dependency on the active site for N and C of bulk CN, NDCN, PVA@NDCN, PAA@NDCN, and PEMAC@NDCN. Note that the symbol ‘+’ defines the NDCN. Free energy diagrams of **c** OER and **d** ORR at *U* = 0.000 V and *U* = 0.402 V for bulk CN, NDCN, and PEMAC@NDCN in alkaline media. **e** Structural deformation induced *sp*^3^-C sites of NDCN and partial density of states (PDOS) of *sp*^2^-C and *sp*^3^-C sites in bulk CN and NDCN, respectively. The Fermi level is indicated by a black dashed line. **f** The optimized structures of NDCN and PEMAC@NDCN with OOH* intermediate at the C5 active site

To further reveal the origin of supramolecular polymer interfaced with N-deficient carbon nitride for OER and ORR activities, the DFT computations are performed to uncover the polymer-incorporated effect on the improved bifunctional activities of the NDCN catalyst. We first considered the single N-defective C_6_N_8_ structure as an N-deficient C_3_N_4_ considering three different corner (NA), center (NB), and bridge (NC) N defect sites in C_3_N_4_ structure as consistent with the controlled experimental characterizations (Fig. S11). Formation energy (*E*_f_) calculations determine the NA-defective C_6_N_8_ (NDCN) is the most promising energetic structure, which displays a similar structure to C_3_N_4_ without severe structural deformation (Fig. S12). Further, we proposed the supramolecular polymers protonated PVA@NDCN, PAA@NDCN, and PEMAC@NDCN structures using polymeric units of polyvinyl alcohol, polyacrylic acid, and poly(ethylene-*alt*-maleic acid), which exhibits polymers interfacing at the carbon and nitrogen sites (Fig. S13). We considered the free energy diagram (FED) approach to calculate the OER/ORR activities by overpotentials (*η*^OER/ORR^) for different structures with/without polymeric bindings (bulk CN, NDCN, PVA@NDCN, PAA@NDCN, and PEMAC@NDCN) for all exposed active sites (Figs. 7a, S12, S14, and S15) [50, 51]. The computed overpotentials for OER/ORR activities (Fig. 7b) show the gradual improvement based on the following order: bulk CN (0.69/0.88 V) > NDCN (0.65/0.66 V) > PVA@NDCN (0.41/0.40 V) > PAA@NDCN (0.36/0.40 V) > PEMAC@NDCN (0.28/0.38 V). This result highlights the crucial role of N deficiency and supramolecular polymer bindings in modifying the reaction potentials to enable OER/ORR bifunctional processes. It can be perceived that the C sites are much more efficient than the N sites due to the electrostatic interactions between positively charged C atoms and anionic intermediate states (O_2_*, O*, OH*, and OOH*) in contrast to the negatively charged N atoms.

The calculated FED results (Fig. 7c, d) reveal that the third step for OER and the first step for ORR are uphill reaction pathways due to the weak binding energy of OOH* species. Further, they are the potential-determining steps for OER and ORR processes (Detailed free energy reactions are shown in supplementary Note S2). Specifically, the binding strengths of OOH* for OER/ORR are notably decreased with N deficiency and supramolecular polymers bindings in the sequence of bulk CN (1.09/0.48 eV) > NDCN (1.05/0.26 eV) > PEMAC@NDCN (0.68/− 0.02 eV). From this result, one can envision that the binding strengths of OOH* play a pivotal role in the determination of OER/ORR activities, and it clearly illustrates the superiority of PEMAC binding. The PEMAC@NDCN performs the lowest overpotentials for OER (0.28 V) and ORR (0.38 V) compared to those of commercial RuO_2_ (0.42 V) and Pt/C (0.45 V) materials, which confirms the PEMAC@NDCN is one of the champion oxygen bifunctional catalysts.

Next, we investigated the geometric and electronic properties of catalysts because adsorbent binding strength is strongly influenced by structural deformation and electrostatic interactions. Following this concept, Fig. 7e (Top) showed that the *sp*^3^-C of NDCN much efficiently facilitates the chemical bindings with OOH* compared to the *sp*^2^-C of bulk CN due to out-of-plane structural deformation [52, 53]. Further, the partial density of states (PDOS, Fig. 7e, bottom) reveals the prominent contribution of *sp*^3^-C from NDCN in the frontier states of Fermi level relative to the *sp*^2^-C from bulk CN, which leads to the activation of catalytic reactions for OER and ORR simultaneously. The NDCN site has a significant influence on the overpotentials compared to bulk CN due to the stabilization of OOH* (Figs. 7f and S16–S17) via electrostatic charge and hydrogen bonding interactions of negatively charged hydroxyl, carboxyl and anhydride groups in the sequence of PVA < PAA < PEMAC [54]. Further, the reduction in bond length between C@NDCN and OOH is associated in the order of 1.461, 1.455, and 1.448 Å for PVA, PAA, and PEMAC, verifying the superiority of OOH* binding strength. In short, supramolecular PEMAC plays significant roles in catalysts as: (1) favorable local environment for OOH* stabilization; (2) regulation of electronic properties for optimal reaction potentials; (3) large surface area with nano-pores promotes the exposure of active sites and mass/ion diffusion; and (4) minimal charge transfer resistances to boost the ORR/OER kinetics.

### Alkaline Zn–Air Batteries

**Fig. 8:**
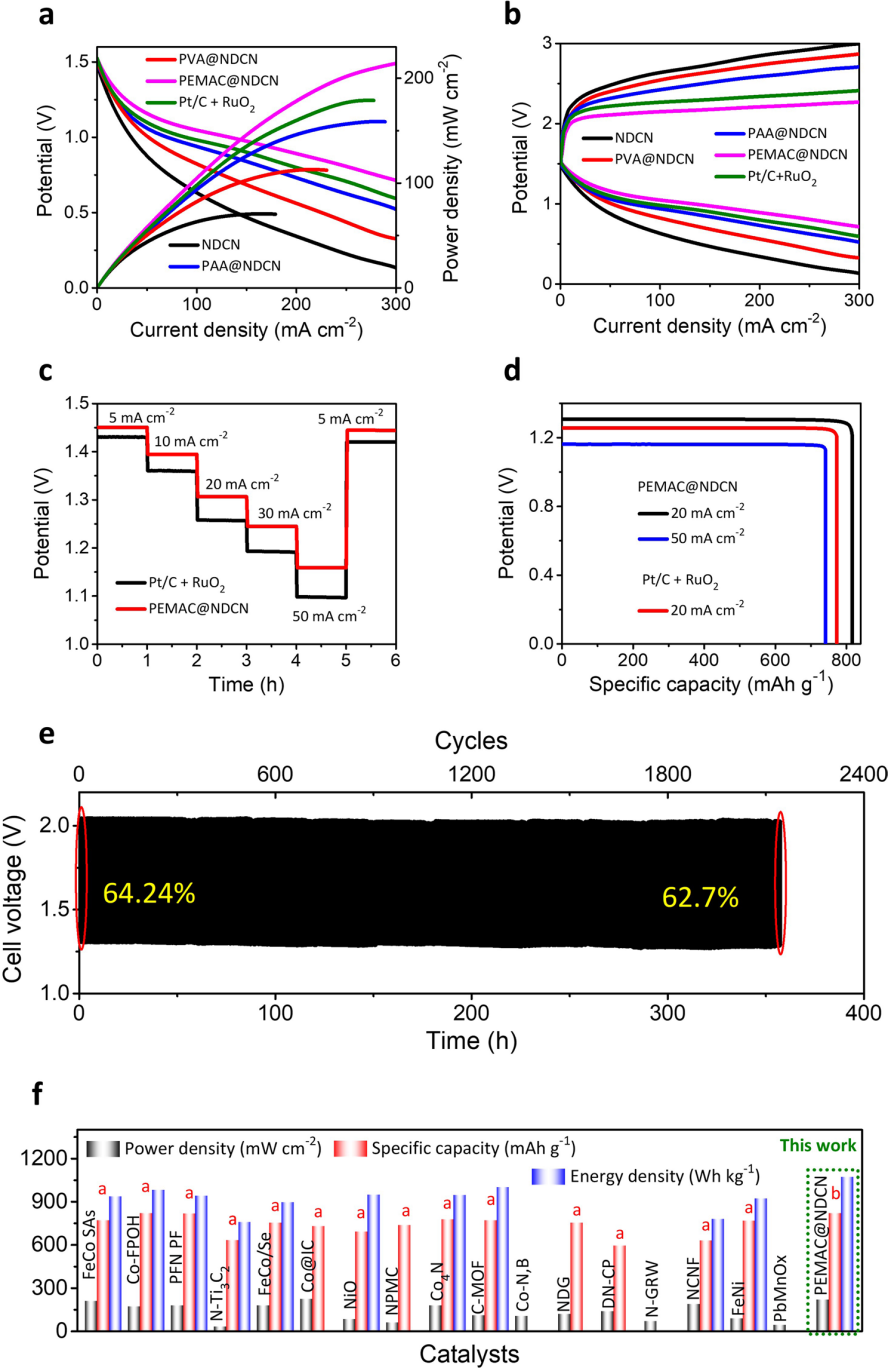
Electrochemical performances for alkaline Zn–air batteries. **a** Power densities and galvanostatic discharge voltage polarizations for ZABs with PEMAC@NDCN, PVA@NDCN, PAA@NDCN, and NDCN cathodes relative to those of Pt/C + RuO_2_ reference materials. **b** Discharge and charge voltage measurements for PEMAC@NDCN relative to those of Pt/C + RuO_2_ reference materials. **c** Rate capacity measurements for PEMAC@NDCN compared to those of Pt/C + RuO_2_ reference materials from 5 to 50 mA cm^−2^. **d** Evaluated capacities and energy densities for PEMAC@NDCN // KOH // Zn and Pt/C + RuO_2_ // KOH // Zn cells. **e** Galvanostatic cycle life evaluations for PEMAC@NDCN // KOH // Zn cells for the current rate of 20 mA cm^−2^ (Time: 10 min per cycle). Displayed percent values are the voltaic efficiencies. **f** Ragone plot presents the projections of capacities and power densities of designed PEMAC@NDCN // KOH // Zn cells in comparison with previously reported champion ZABs. (Conditions: cathode- PEMAC free-standing air cathode, electrolyte-6 M KOH + 0.2 M Zn acetate, and anode-Zn plate)

Considering the champion oxygen bifunctional performances, we constructed the prototype alkaline liquid ZABs consisting of a supramolecular gel derived PEMAC@NDCN membrane as a flexible free-standing air cathode, Zn foil as anode, and alkaline liquid electrolyte (KOH, 6 M + Zn(OAc)_2_, 0.2 M, Fig. S18). The ZABs with PEMAC@NDCN free-standing cathode displays a superb power density of 214 mW cm^−2^ (Fig. 8a), which surpasses the reference Pt/C + RuO_2_ materials (178 mW cm^−2^) and majority of the reported bifunctional cathodes in alkaline ZABs (Table S5, Supplementary Information). Figure 8b displays the PEMAC@NDCN ZABs possess the lowest overpotentials (corresponds to the highest energy efficiencies) for a wide range of current densities during galvanostatic discharge and charge states compared to those of other prepared and benchmark Pt/C + RuO_2_ catalysts. Galvanostatic discharge profiles (Fig. 8c) display the excellent rate-capability for the current densities of 5–50 mA cm^−2^ with discharge potentials 1.43–1.16 V and vice versa while reference Pt/C + RuO_2_ decays the discharge potential during recovery states, reflecting the outstanding efficiency and reversibility of PEMAC@NDCN ZABs. The ZABs with PEMAC@NDCN determine the highest specific capacity of 817 mAh g_Zn_^−1^ at 20 mA cm^−2^ compared to those of Pt/C + RuO_2_ (773 mAh g_Zn_^−1^, Fig. 8d). Furthermore, it also retains the 742 mAh g_Zn_^−1^ capacity even for the high current density of 50 mA cm^−2^. Furthermore, we achieved the highest gravimetric energy densities of 1070 Wh kg_Zn_^−1^ for PEMAC@NDCN, which outperforms the reference Pt/C + RuO_2_ (966 Wh kg_Zn_^−1^). The long-lasting cycle life of alkaline liquid-state ZABs with PEMAC@NDCN membrane air cathodes is determined by measuring galvanostatic discharge and charge tests for a harsh current density of 20 mA cm^−2^. It exhibits superb energy efficiency of 64.24% and discharge–charge cycling operations over 2160 cycles (~ 360 h) with negligible decay compared to those of reference Pt/C + RuO_2_ materials (240 cycles for 40 h with 21.2%) and most of the champion alkaline ZABs reported to date under harsh conditions, indicating the unprecedented operational durability (Figs. 8e, S19 and Tables S5–S6). PEMAC@NDCN delivers the highest specific capacities, power, and energy densities under harsh operating conditions ever reported so far for the alkaline ZABs (Fig. 8f and Tables S5–S6) [1–49]. Post-cycling structural, morphology, and chemical characterizations of PEMAC@NDCN catalysts after operations over 2160 cycles have been displayed in Figs. S20–S22, Supplementary Information. XRD pattern, SEM, and HRTEM images show the well-defined hexagonal crystal structure with interconnected polymer interfaced nanotubes frameworks. HRTEM image reveals the existence of definite polymeric dispersion over NDCN without noticeable destruction. Furthermore, XPS characterization also maintains their initial chemical oxidation states and spin–orbit splittings of C, N, and O, which clearly illustrates the robustness of the designed PEMAC@NDCN catalysts. In addition, the morphology evaluation of Zn anode after 2160 cycles is shown in Fig. S23. SEM image displays a highly rough surface with formation of ZnO and severe consumption of Zn due to the corrosion reactions in alkaline electrolyte; however, no obvious dendrites structures demonstrate the superiority of PEMAC@NDCN flexible cathodes.

### Solid-State Zn–Air Batteries

**Fig. 9:**
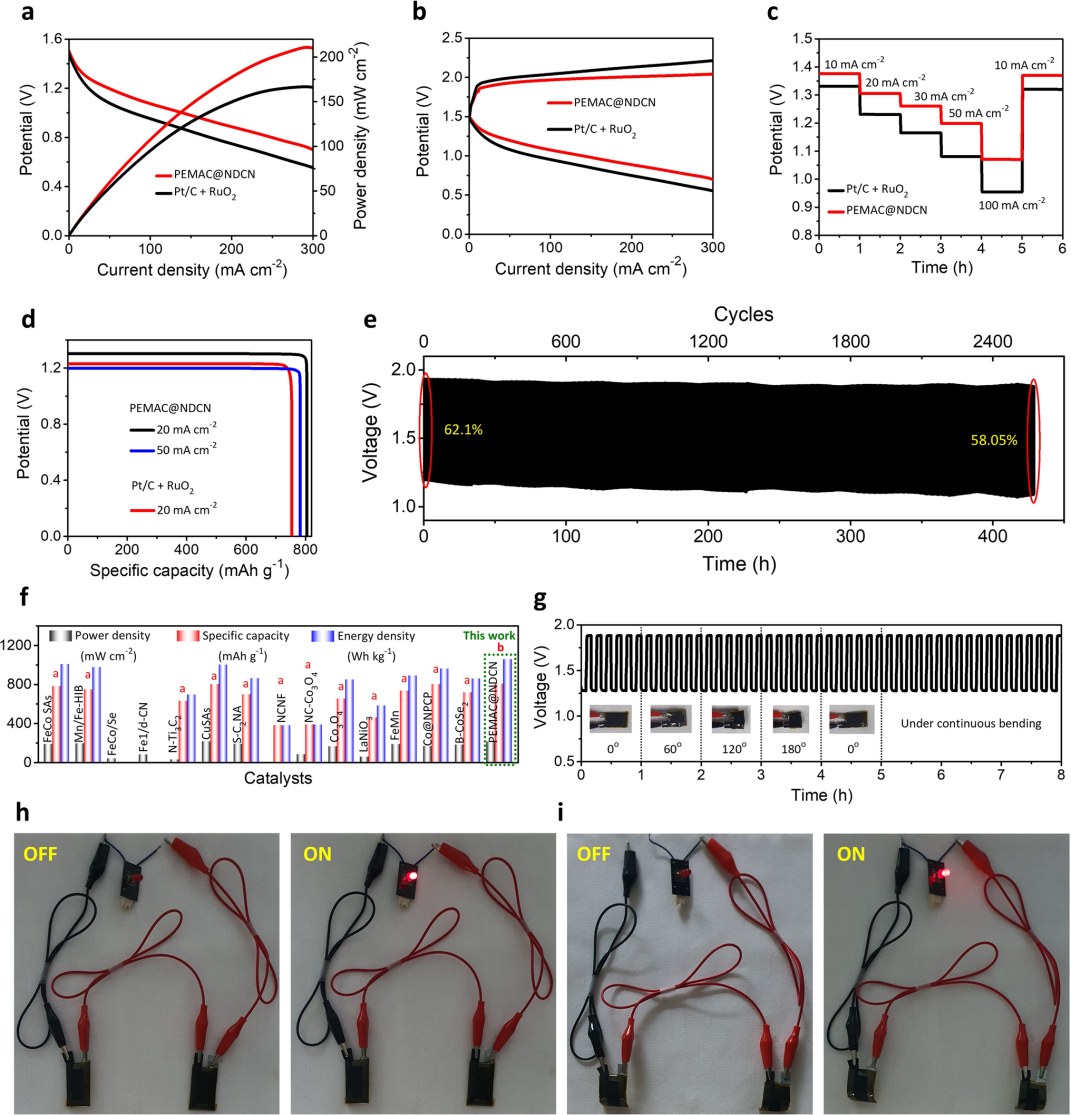
Electrochemical performances for flexible ZABs. **a** Power densities and galvanostatic discharge voltage polarizations for PEMAC@NDCN // CBCs // Zn compared to those of reference Pt/C + RuO_2_ // CBCs // Zn cells. **b** Discharge and charge measurements for PEMAC@NDCN relative to Pt/C + RuO_2_ reference materials. **c** Rate capacity measurements for PEMAC@NDCN // CBCs // Zn relative to those of reference Pt/C + RuO_2_ // CBCs // Zn cells from 10 to 100 mA cm^−2^. **d** Calculated capacities and energy densities for PEMAC@NDCN // CBCs // Zn and Pt/C + RuO_2_ // CBCs // Zn cells. **e** Cycle life assessments for PEMAC@NDCN // CBCs // Zn cells for the current rate of 50 mA cm^−2^ (Time: 10 min per cycle). Note that the stated percent values are the voltaic efficiencies. **f** Ragone plot illustrates the projections over cell capacities and energy densities of designed PEMAC@NDCN // CBCs // Zn cells in comparison with previously reported champion ZABs. Note that the “a” defines the current density in the range of 1–5 mA cm^−2^ and “b” represents the current density in the range of 25–50 mA cm^−2^. **g** Discharge–charge mechanical bent testing (0, 60, 120, and 180°) and with continuous bent over 60° of PEMAC@NDCN // CBCs // Zn cells for 25 mA cm^−2^. Inset shows the practical pictures of ZABs. **h** Powering of red LED using two ZABs in series under planar conditions. **i** Powering of red LED using two ZABs in series under bent condition. (Conditions: cathode-PEMAC punched on carbon cloth, electrolyte-CBCs^1^, and anode-Zn plate)

Inspired by flexible free-standing features of guanosine supramolecular gel derived PEMAC@NDCN membrane and growing demands for wearable technologies, the prototype solid-state ZABs have been constructed using the straightforwardly PEMAC@NDCN membrane as air electrode, anti-freezing chitosan biocellulosics membrane as electrolyte (CBCs [1]), and pristine Zn foil as anode (Fig. S24). For comparison, the ZABs with reference Pt/C + RuO_2_ materials (1:1 w/w) are also fabricated. Flexible ZABs display a stable open circuit potential (OCP) of ~ 1.5 V (Fig. S25) and superb power density of 211 mW cm^−2^ compared to those of reference Pt/C + RuO_2_ derived ZABs (~ 1.48 V, 166 mW cm^−2^, Fig. 9a) and reported solid-state ZABs (Table S7). Galvanostatic charge and discharge states (Fig. 9b) for the PEMAC@NDCN based flexible ZABs reveal the lowest charge–discharge overpotentials and highest energy efficiencies compared to those of Pt/C + RuO_2_ reference ZABs for wider windows of the current densities from 0 to 300 mA cm^−2^. ZABs with PEMAC@NDCN indicate higher discharge voltage plateaus than those of Pt/C + RuO_2_ for the hierarchy of current densities from 10 to 100 mA cm^−2^ (Fig. 9c), two times higher than the alkaline liquid ZABs (Fig. 8c), which demonstrates the excellent rate characteristics due to the superior ORR kinetics and great prospective for the realistic implementations. In addition, the stable discharge rate properties, specifically under large current densities, confirm the ZABs operations reliability over extremely harsh operating conditions. Furthermore, the PEMAC@NDCN ZABs obtain the specific capacity of 806 and 782 mAh g^−1^ for the current densities of 20 and 50 mA cm^−2^, surpassing those of reference Pt/C + RuO_2_ (753 mAh g^−1^, Fig. 9d) and previous reports. The PEMAC@NDCN delivers a high capacity even for 50 mA cm^−2^, illustrating the promising pathways for hash operations. The determined energy densities of PEMAC@NDCN are 1056 and 938.4 Wh kg^−1^ for 20 and 50 mA cm^−2^ current densities, which outperforms the commercial Pt/C + RuO_2_ (926 Wh kg^−1^) and recently reported champion flexible ZABs (Fig. 9d and Table S7). For comparison, the cell-level discharge capacity of 83 mAh g_cell_^−1^, the energy density of 108 Wh kg_cell_^−1^ at 20 mA cm^−2^, and power density of 441 W kg_cell_^−1^ (including all active and inactive materials of a cell) are recorded. The volumetric capacity and energy density are 400 mAh cm^−3^ and 524 Wh L^−1^, respectively. Moreover, ZAB withstood more than 2580 charge–discharge cycles (~ 430 h) for a 50 mA cm^−2^ current density while maintaining stable reversible capability (Fig. 9e). This far surpasses precious Pt/C + RuO_2_ reference materials (360 cycles for 60 h) and most reported ZABs catalysts (Fig. S26 and Table S7). As shown in Fig. S27, the ZABs are capable of stable operations over higher charge and discharge states. Most of the previously reported ZABs performances (specific capacity, energy density, and cycle life) are evaluated based on the lower current densities or current rates with alkaline liquid (1–10 mA cm^−2^) and solid-state (1–2 mA cm^−2^) electrolytes with shallow charge–discharge states, which underperforms compared to those of the realistic scale commercial batteries (e.g., LIBs). Recently, Shinde et al. [1] demonstrated the measurements of solid-state ZABs under realistic, practical conditions. In light of these facts, it is necessary to measure ZAB performance at least for higher current density as one of the factors to minimize the practical limitations of ZABs. This work demonstrates superb ZABs performances in terms of power density, specific capacity, energy density, and cycle life with the ability to withstand harsh operation (Fig. 9f and Table S7) [1–5, 7–49, 55]. Upon mechanical bendings from 0° to 180° and vice versa, the solid-state ZABs possess stable charge–discharge operations and galvanostatic cycling over a wide range of current densities (Figs. 9g and S28), which indicates the outstanding electrochemical and mechanical durability with significant prospects for flexible technologies. The practical application of solid-state ZABs with PEMAC@NDCN effectively displayed the operations for red light emitting diode with serial connections of two ZABs (Fig. 9h, i) under planar and bent states, which suggests the outlook for commercial wearable electronics technology. Post-mortem analysis reveals the retention of structural, morphology, elemental maps, and chemical properties comparable to pristine PEMAC@NDCN membrane catalysts (Figs. S29–S31). Furthermore, Zn anode also has a smooth surface without the formation of dendrites (Fig. S32). This feature implies the robust structures of PEMAC@NDCN and compatibility with electrolytes and Zn anodes.

### All-temperature Zn–Air Batteries

**Fig. 10:**
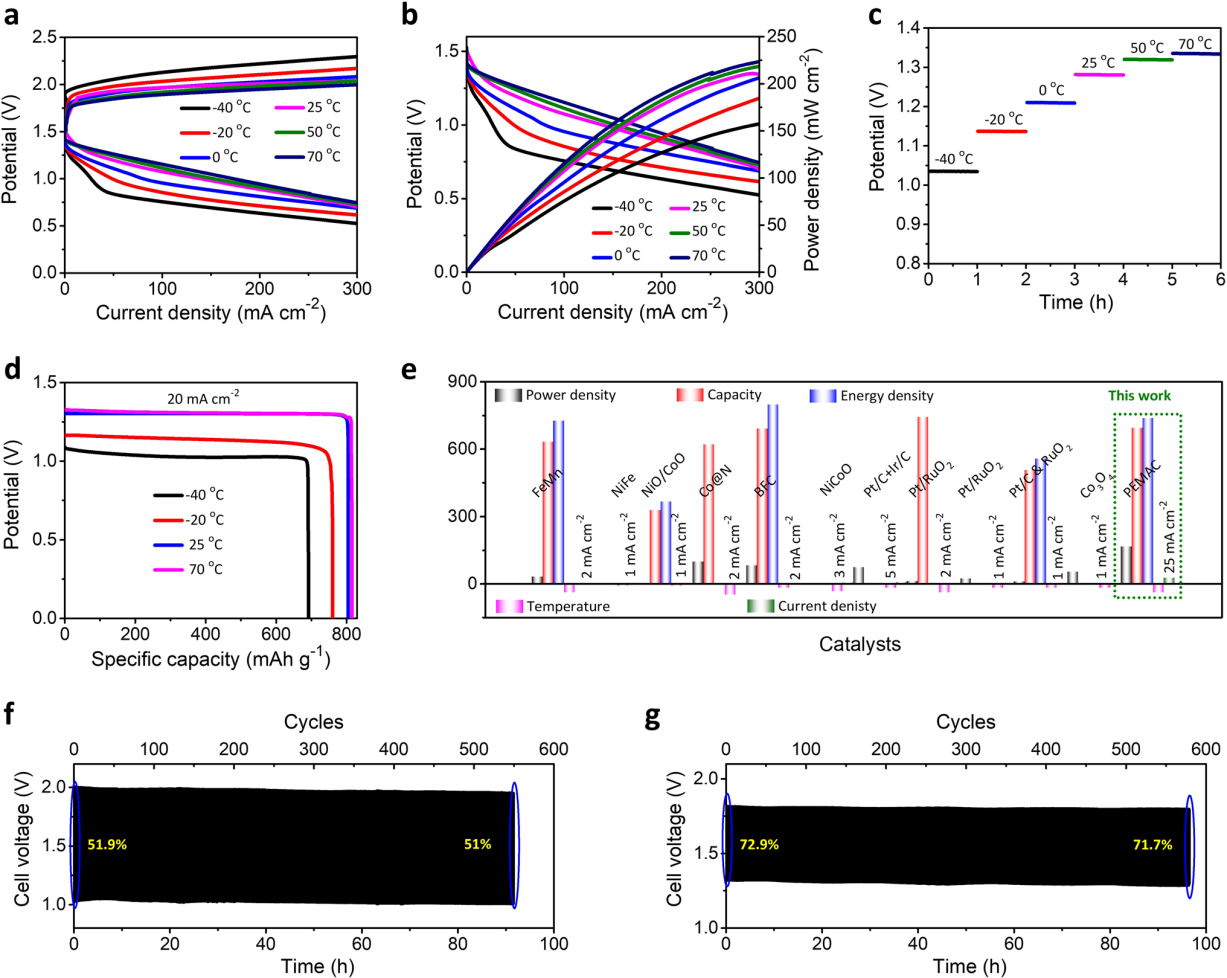
All temperature range electrochemical performances of flexible ZABs. **a** Discharge and charge measurements for PEMAC@NDCN // CBCs // Zn for wide temperatures from − 40 to 70 °C. **b** Power densities and galvanostatic discharge voltage polarizations for PEMAC@NDCN // CBCs // Zn for wide range of temperatures from − 40 to 70 °C. **c** Discharge potentials measurement over wide temperatures at 25 mA cm^−2^. **d** Calculated capacities and energy densities for PEMAC@NDCN // CBCs // Zn for − 40, − 20, 25, and 70 °C at 20 mA cm^−2^. **e** Ragone plot illustrates the projections over performances retentions (i.e., in terms of capacity (mAh g^−1^), power (mW cm^−2^), energy (Wh kg^−1^), temperature (°C), and current densities (mA cm^−2^) of designed PEMAC@NDCN // CBCs // Zn cells in comparison with previously reported champion ZABs for the low-temperatures region. Note that all the reported ZABs performances are 1–5 mA cm^−2^, and the measurements for this work are taken at 20–25 mA cm^−2^. **f** and **g** Cycle life for PEMAC@NDCN // CBCs // Zn cells for the current rate of 25 mA cm^−2^ at − 40 °C and 70 °C. Time: 10 min per cycle

We further evaluated the ZABs operations for wide temperatures of − 40 ~ 70 °C to determine the tolerance against extreme environmental conditions (Fig. 10). Notably, ZABs display superb electrochemical performances to withstand wide temperatures from − 40 to 70 °C in terms of capacity, power, energy, and cycle life. For high temperatures of 70 °C, the ZABs deliver the enhancement in the OCP to 1.53 V and higher energy efficiencies over wide range of current densities compared to those of room temperature (RT) values as verified by galvanostatic polarizations. For an ultra-low temperature of − 40 °C, the ZABs retain the OCP of 1.46 V (96%) and remarkable energy efficiencies over higher polarizations comparable or slightly lower than that of the RT counterpart (Fig. 10a). ZABs still maintain the power density of 159 mW cm^−2^ (75.4%) when the temperature drops to − 40 °C, while it increases to the 224 mW cm^−2^ (106%) for higher temperature of 70 °C, compared to those of RT values and most of the previous reports of ZABs; note that typical ZABs scarcely work over the ultra-low temperature of − 40 °C revealing severe degradation of power densities (> 70–80%) (Fig. 10b and Table S8). Furthermore, ZAB-PEMAC@NDCN shows superb discharge voltage plateaus with extremely harsh operating environments from − 40 to 70 °C for 25 mA cm^−2^ current density compared to ZAB-Pt/C + RuO_2_ (Fig. 10c), illustrating a superior rate capability. As shown in Fig. 10d, ZAB-PEMAC@NDCN delivers a specific capacity of 817 mAh g^−1^ and energy density of 1074 Wh kg^−1^ for a higher temperature of 70 °C at 20 mA cm^−2^, which corresponds to the 102% preservation relative to those of RT counterparts (806 mAh g^−1^ and 1056 Wh kg^−1^). While for the ultra-low temperature of − 40 °C, ZABs still retain the capacity of 693 mAh g^−1^ (86%) and energy density of 738 Wh kg^−1^ (~ 70%). Ragone plots (Fig. 10e and Table S8) reveal the superb environmental durability of ZABs for both ultra-low and high-temperature conditions with retention of champion capacity, energy density, power density, and cycling life even for the harsh applied current densities (20–25 mA cm^−2^) compared to those of previously reported ZABs so far (1–5 mA cm^−2^), illustrating the superb electrochemical performance of ZABs under harsh operational conditions [1–5, 7–12, 15–21, 23–25, 27, 36, 37, 42, 55]. ZAB-PEMAC@NDCN reveals the stable discharge–charge electrochemical cycling performance over ~ 550 cycles (~ 91.5 h, 51.9%) and ~ 580 cycles (~ 96.4 h, 72.9%) for − 40 and 70 °C at 25 mA cm^−2^ current density with negligible voltage efficiency decays, which demonstrates the superior, long-lasting durability for harsh operational conditions (Fig. 10f, g). The broad temperature flexibility of PEMAC@NDCN ZABs is attributed to the followings. (1) The electronic coupling of supramolecular polymers with 1D-NDCN frameworks offers a large specific surface area with abundant active sites fully exposed to the surface (i.e., transport channels for fast reactants permeation), which reduces the ion diffusion distance and confirms the exceptional admittance for ions to the active sites, diminishing the negative influence for hindered ion diffusion kinetics. (2) Strong *π*–*π* interactions between polymer species and NDCN anchor the critical bridge for interlayered chain structures, thus increasing the binding energy. These multiple interactions retain the anti-drying and anti-freezing properties for wide temperatures. (3) Supramolecular polymers interact strongly with water molecules forming abundant hydrogen bonding, which aims to reinforce the interactions with electrolyte networks. (4) Synergistic influence of supramolecular polymers reduces the activation energy barrier of PEMAC@NDCN catalysts, which outcomes the impervious reaction kinetics. (5) The strong adhesion of KOH exchanged CBCs electrolyte with PEMAC@NDCN enables conformal electrical functioning with electrolyte interface and air electrode, demonstrating a critical aspect for operations of wearable electronics. More interestingly, these results significantly outperform the previous reports for anti-freezing ZABs, which vindicates the prospects of ZABs for harsh operating conditions at all temperatures, confirming the unprecedented outcomes.

## Conclusions

In summary, we present a facile approach for the rational design of supramolecular polymers intertwined N-deficient carbon nitride nanotubes frameworks as 3D flexible free-standing membrane catalysts via bottom-up self-conversion reactions. The PEMAC@NDCN catalysts display superb bifunctional (ORR and OER) performances and durability, surpassing those of reference Pt/C, RuO_2_, and the most reported champion catalysts, owing to the hierarchy of porous frameworks with optimal interfaced configurations and abundant active sites. Furthermore, the alkaline liquid-state ZABs with free-standing cathode reveal outstanding reversibility and cycle life which are superior to most reported liquid ZABs. Notably, the flexible solid-state ZABs exhibit stable discharge–charge rechargeability for 2580 cycles at 50 mA cm^−2^, specific capacity of 806 mAh g^−1^, energy density of 1056 Wh kg^−1^, stable mechanical durability, and wide temperature operations from − 40 to 70 °C for 20–50 mA cm^−2^ applied current densities. The theoretical and experimental results demonstrate insights for engineering the superb free-standing catalysts via boosting intrinsic activity and transport capabilities, providing a feasible strategy for fabrication and thereby promising the reliability of flexible ZABs under harsh operating environmental conditions.

## Supplementary Information

Below is the link to the electronic supplementary material.

Supplementary file1 (PDF 2484 KB)
