# Supplementary material for: Supramolecular Polymer Intertwined Free-Standing Bifunctional Membrane Catalysts for All-Temperature Flexible Zn–Air Batteries
Source: Nanomicro Lett. 2022 Sep 17;14:190. doi: 10.1007/s40820-022-00927-0 (PMC9482563; doi:10.1007/s40820-022-00927-0)
Supplement: Supplementary file 1 — Supplementary file1 (PDF 2484 KB) [file 40820_2022_927_MOESM1_ESM.pdf]

Supporting Information for

Supramolecular-Polymer Intertwined Free-Standing Bifunctional Membrane Catalysts for All-Temperature Flexible Zn-Air Batteries

Nayantara K. Wagh[1, #], Sambhaji S. Shinde[1, #], Chi Ho Lee[2], Sung-Hae Kim[1], Dong-Hyung Kim[1], Han-Don Um[3], Sang Uck Lee[2, *], Jung-Ho Lee[1, *]

[1]Department of Materials Science and Chemical Engineering, Hanyang University, Ansan, Republic of Korea

[2]Departament of Applied Chemistry, Center for Bionano Intelligence Education and Research, Hanyang University, Ansan, Republic of Korea

[3]Departament of Chemical Engineering, Kangwon National University, Chuncheon, Gangwon 24341, Republic of Korea

*Corresponding authors. E-mail: jungho@hanyang.ac.kr (JHL); sulee@hanyang.ac.kr (SUL)

S1 Note S1: Experimental Methods

S1.1 Catalyst Preparation

Chemicals

Melamine ($C_3H_6N_6$), polyvinyl alcohol ($(C_2H_4O)_x$), polyacrylic acid ($(C_3H_4O_2)_n$), Poly(ethylene-alt-maleic anhydride) (Mw 100,000-500,000), ammonium thiosulfate, guanosine, and nafion were obtained with the Sigma-Aldrich. Ethylene glycol ($C_2H_6O_2$), ethanol (C_2H_5OH), acetone, potassium hydroxide (KOH), sulfuric acid (H_2SO_4), zinc acetate ($Zn(OAc)_2$), and nitric acid (HNO_3) were attained from Daejung Chemicals. Carbon paper and carbon cloth (Spectra carb 2040-A, Fuel Cell Store), and Zn plate (Sigma-Aldrich, USA) were used as received. All precursors were used without further purifications. Deionized water was utilized as solvent until otherwise specifically mentioned. Received resources were applied further without distillation.

Synthesis of reference Pt/C catalysts

Poly(N-vinyl-2-pyrrolidone) (M_W = 40,000) was treated with an appropriate amount of chloroplatinic acid ($H_2PtCl_6 \cdot 6H_2O$) solution under vigorous stirring, followed by the addition of activated carbon. After that, the sodium borohydride ($NaBH_4$) solution was appended to the reaction mixture and kept under stirring for another 30 min. Finally, the obtained mixture was collected after centrifugation and several cleanings with deionized water and dried at 60 °C for 12 h.

Synthesis of the RuO_2 catalysts

The ruthenium (III) chloride hydrate (50 mM) was dispersed in the mixture of methyl alcohol and water (1:1 v/v) under vigorous stirring. Further, the sodium hydroxide (NaOH, 2 M) was inserted drop wisely in the above reaction mixture until the pH of the solution became 7. The product was centrifuged, rinsed with deionized water, and dehydrated for 60 °C, followed by annealing at 500 °C for 5 h in air.

S1.2 Characterization of the Prepared Catalysts

Powder X-ray diffraction (PXRD) patterns were measured on Rigaku Smartlab D/max 2500Pc diffractometer with Cu Kα radiation (wavelength of 1.5406 Å). Morphology images were performed by field-emission scanning electron microscopy (FESEM, JEOL-6700F). Transmission electron microscopy (TEM) images were acquired on a Cs-corrected Titan™

80-300 with an accelerating voltage of 80 kV. Elemental composition maps were recorded by an EDS attached to the TEM. FTIR measurements were performed on a Perkin Elmer GX spectrometer. The nitrogen sorption measurements were conducted on an AUTOSORB-1-MP surface area and pore size analyzer using a liquid nitrogen bath (77 K). The surface volume, area, and pore profiles were analyzed from the adsorption and desorption branches by the Brunauer-Emmett-Teller (BET) and Barrett−Joyner−Halenda (BJH) methods. X-ray photoelectron spectroscopy (XPS) was performed on a VG SCIENTA (R 3000) spectrometer equipped with a monochromatic Al *K*α source.

S1.3 Electrochemical Measurements

The electrochemical performances were performed on an electrochemical workstation (CHI 760 D, CH Instruments) at room temperature with a typical three-electrode electrochemical cell. The Ag/AgCl (with saturated KCl) reference electrode, a graphite rod as the counter electrode, a glassy carbon (GC) rotating disk electrode (RDE, 0.196 cm^2) coated with the catalysts as the working electrode, and 0.1 M aqueous KOH (oxygen-rich) as the electrolyte. The working electrode was fabricated with as-obtained PEMAC@NDCN or PVA@NDCN or PAA@NDCN catalysts punched over the GC using nafion adhesive (5 wt%, 2 μL) and then dried at room temperature for several hours. For comparison, Pt/C and RuO_2 reference materials inks were prepared by dispersing 10 mg of catalysts in the propanol of 1.95 ml and nafion of 50 μL. Then, 6 μL of the prepared inks were evenly loaded on the GC surface and dried for room temperature. Mass loading for both fabricated catalysts was fixed to be 0.15 mg cm^{-2} for both oxygen reactions. Prior to the ORR/OER measurements, the electrolyte (0.1 M KOH) was saturated by oxygen, nitrogen flow for 30 min. CVs were performed in O_2 and N_2-saturated electrolyte with a scan rate of 50 mV s^{-1}. LSV profiles for ORR/OER were obtained for 1600 rpm with a scan rate of 5 mV s^{-1} in the potential range of 0.2-2 V vs. RHE. Chronoamperometric half-cell reactions durability was evaluated at the respective overpotentials for OER and half-wave potential for ORR. The measured potentials were referenced to the reversible hydrogen electrode (RHE) according to the Nernst equation,

$$E_{RHE} = E_{Ag/Ag/Cl} + 0.059 \times pH + 0.205 \qquad \text{(S1)}$$

Performed current densities were also referenced according to the measured geometric surface areas. Electrochemical impedance spectra were obtained for the frequency range of 100 Hz to 1 MHz with a constant bias of 0.2 V. The long-life durability of the catalysts was measured by continuous potentiodynamic sweeps for a scan rate of 100 mV s^{-1}. The mass loading of membrane catalysts and reference Pt/C/RuO_2 has been placed identically unless otherwise stated (0.15 mg cm^{-2}).

S1.4 Oxygen Reduction Reaction Kinetics

The ORR kinetics were evaluated by using different rotational speed LSV profiles. The measured total current density is the sum of inverse of kinetic current (J_K) and diffusion current (J_d). Every atom or ion on the electrode reacts immediately as the applied overpotential is sufficiently high. The number of oxygen molecules at the electrode surface is almost zero, facilitating diffusion-limiting plateau. Therefore, the diffusion current is related only to the RDE rotational speeds.

The transferred electron number (n) in oxygen reduction was determined according to the Koutecky-Levich (K-L) equation [S1-S3]:

$$\frac{1}{J} = \frac{1}{J_K} + \frac{1}{B\omega^{1/2}} \qquad \text{(S2)}$$

$$B = 0.2nF(D_{O_2})^{2/3}\nu^{-1/6}C_{O_2} \quad (S3)$$

where B represents the Levich slope, J_K represents the kinetic current, J represents the measured total current, ω represents the electrode rotation rate, n represents the number of electrons transferred for each oxygen molecule, F represents the Faraday constant (F = 96485 C mol^{-1}), D_{O2} represents the O_2 diffusion coefficient in 0.1 M KOH (D_{O2} = 1.9×10^{-5} cm^2 s^{-1}), ν represents the kinetic viscosity (0.01 cm^2 s^{-1}) and C_{O2} represents the concentration of O_2 (C_{O2} = 1.2 $\times10^{-6}$ mol^{-1} cm^{-3}). The considered rotation speeds are in rpm, and therefore, the constant factor 0.2 is multiplied. The peroxide species during ORR reactions were determined by measuring the RRDE polarization profiles for the ring potential of 1.3 V *vs.* RHE. Based on the following expressions, the transferred electron number (n) and peroxide (H_2O_2) yield were evaluated as [S4]:

$$n = 4\frac{I_d}{I_d + I_r/N} \quad (S4)$$

$$H_2O^-(\%) = 100\frac{2I_r/N}{I_d + I_r/N} \quad (S5)$$

where I_r represents the ring current, I_d represents the disk current, and N represents the current collection efficiency of the Pt ring. N was determined to be 0.42.

S1.5 Fabrication of Zn-air Batteries

Alkaline Zn-air batteries

The rechargeable Zn-air battery performance was analyzed on home-built electrochemical cells. Air cathodes were constructed by uniform punching of PEMAC@NDCN membrane (1 cm × 1 cm) and carbon paper was utilized as current collectors. Further, for comparison, Pt/C + RuO_2 catalyst slurry was fabricated by mixing carbon black, polytetrafluoroethylene, and the catalysts (1:1:8 w/w) in ethanol/Nafion solution. The mass loadings of the PEMAC@NDCN and reference Pt/C+RuO_2 catalysts for rechargeable ZABs was 1 mg cm^{-2}. Mass ratio of reference Pt/C and RuO_2 was of 1:1. Here, 6 M KOH and 0.2 M zinc acetate was used as the electrolyte for the reversible electrochemical reactions. Catalyst-loaded (Pt/C+RuO_2) carbon paper was used as an air cathode and polished Zn plate (0.5 mm thickness) as anode.

Flexible solid-state Zn-air batteries

Flexible Zn-air batteries were fabricated by including PEMAC@NDCN membrane catalysts as the air cathodes, the chitosan biocellulosics (CBCs fabricated as per our previous reference[1]) as the solid electrolyte (50 μm in thickness), and Zn foil (0.3 mm) as the anode. Then, membrane catalysts and zinc foils were positioned on opposite sides of the bio-cellulose membrane electrolyte, without any external current collector. Finally, the assembled devices were pressed cautiously and encapsulated with sustainable latex. For comparison, Pt/C + RuO_2 catalyst slurry was also fabricated by mixing carbon black, polytetrafluoroethylene, and the catalysts (1:1:8 w/w) in ethanol/Nafion solution. The mass loadings of the PEMAC@NDCN and reference Pt/C+RuO_2 catalysts for rechargeable ZABs was 2 mg cm^{-2} and electrode area of 2 cm^2.

Battery testing

All the fabricated ZABs were evaluated under atmospheric conditions. The galvanostatic discharge and charge voltage profiles were conducted on a LAND CT2001A multichannel

battery testing system. The cycling for alkaline and flexible solid ZABs was performed for 10 min per cycle (discharge: 5 min; charge: 5 min) with 20 and 50 mA cm^{-2} current density, respectively. The specific capacities were determined using the galvanostatic discharge profiles standardized to the consumed mass of Zn. The energy efficiency was calculated from the ratio of discharge to charge voltages. The power densities of both ZABs were calculated by expression as $P = V \times I$.

S2 Supplementary Figures

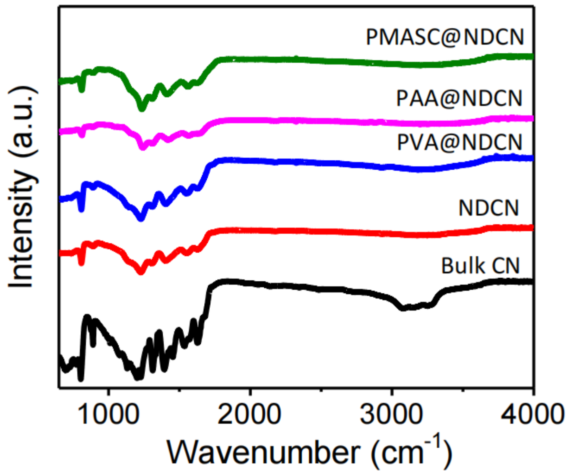

Fig. S1 Spectroscopic characterizations. Fourier transforms IR spectra for PEMAC@NDCN compared to those of PVA@NDCN, PAA@NDCN, NDCN, and bulk CN

The molecular binding structures for PEMAC@NDCN compared to those of PVA@NDCN, PAA@NDCN, NDCN, and bulk CN are evaluated by FTIR spectra. The characteristic s-triazine ring structure breathing mode was observed for 800-891 cm^{-1} for bulk CN, which illustrates carbon nitride frameworks [S5]. Furthermore, the decreasing intensity of s-triazine peaks for NDCN, PEMAC@NDCN, PVA@NDCN, and PAA@NDCN confirms the formation of nitrogen deficiency and loading of polymeric layers. The peaks from 1100-1600 cm^{-1} are attributed to the stretching bindings of C-N, C=C, and C=N, which demonstrates the existence of pyridinic, pyrrolic, and graphitic nitrogen species, as evident with XPS N 1s spectra [S6]. Characteristic broad bindings over 3000-3500 cm^{-1} corresponds to the C=C-H, C-H, stretch, amine or water bindings [S7]. Presence of electron-donating species such as ortho, para and meta phases in polymer-based NDCN structures, which suggests the enhancement for charge transfer during electrochemical reactions. The stretching vibrations for 1418 and 1327 cm^{-1} were observed conforming to the anti-symmetric and symmetric carboxylic anions and C=C, C=O, C-H bond structures, which indicates the formation of PEMAC@NDCN.S8 PEMAC@NDCN, PVA@NDCN, and PAA@NDCN illustrates the weak binding for ~2181 cm^{-1}, which clarifies the stretching vibrations for N=C=N and N=C=O. This illustrates the presence of pyridinic $N^{+}O^{-}$ species as evident in XPS results [S9]. The variation in the C-N or C=C stretching bindings compared to those bulk CN illustrates the construction of polymeric CN structures. The presence of peaks at 1632 and 1689 cm^{-1} corresponds to the formation of C-O or C-O-H groups from the polymeric chain. The existence of C=C, C=O, OH, C-H illustrates the negatively charged polymeric moieties attached to the surface of NDCN, which is critical for enhancing electrochemical kinetics. FTIR characterization revealed the formation of polymer-derived metal-free NDCN frameworks.

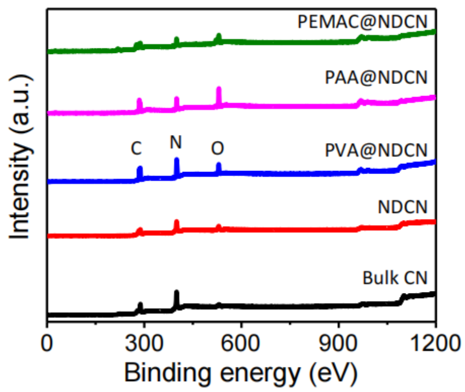

Fig. S2 Spectroscopic characterizations. Full scan XPS spectra for designed polymeric PEMAC@NDCN, compared to those of PVA@NDCN, PAA@NDCN, NDCN, and bulk CN catalysts. Note that the representative C, N, and O are observed without further impurities, clarifying the formation of polymeric catalysts

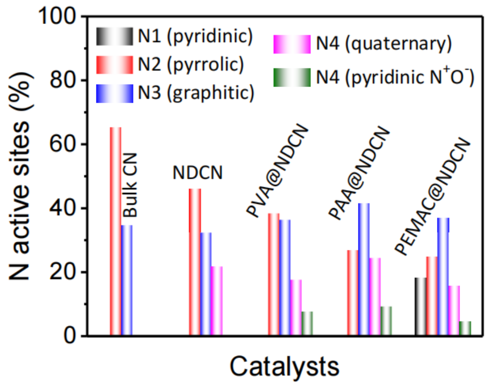

Fig. S3 Spectroscopic characterizations. Quantitative illustrations for the obtained nitrogen species over various polymeric and CN catalysts

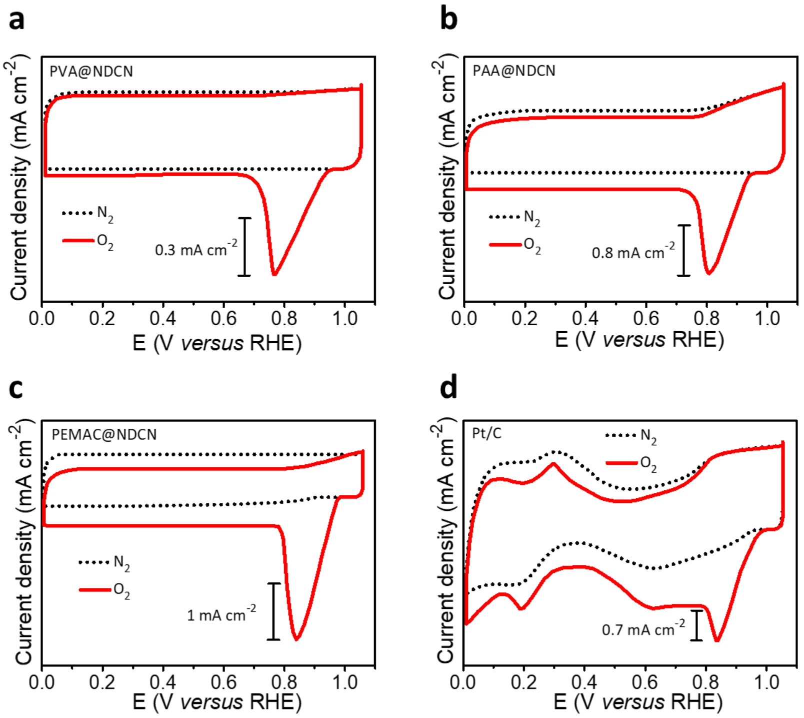

Fig. S4 Electrochemical performance. CVs scans over N_2- (black dots) and O_2-saturated (red solid) for (**a**) PVA@NDCN, (**b**) PAA@NDCN, (**c**) PEMAC@NDCN, and (**d**) Pt/C catalysts in the 0.1 M KOH electrolyte solutions. Scan rate 50 mV s^{-1}

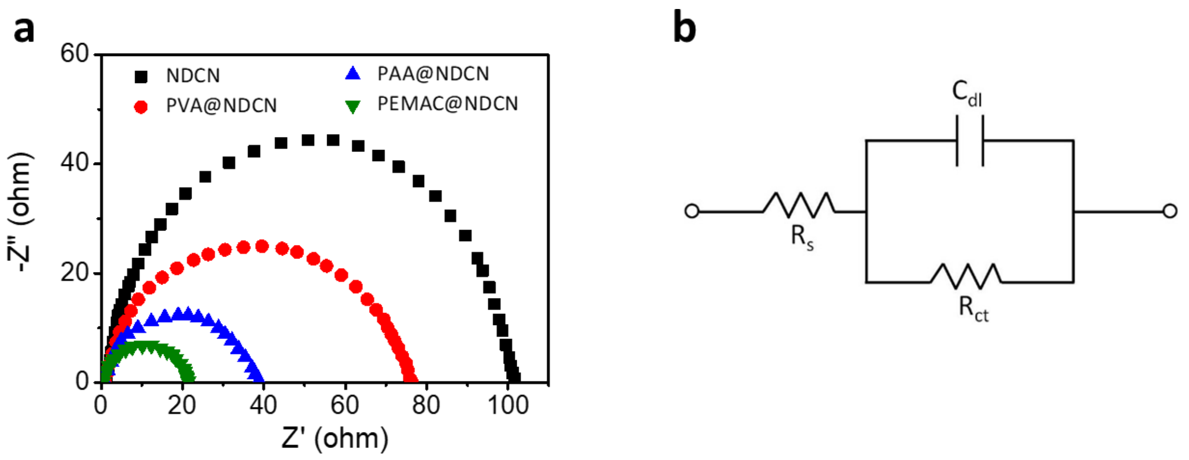

Fig. S5 Electrochemical kinetics for ORR. (**a**) Electrochemical impedance spectra (EIS) for fabricated polymeric and CN catalysts during the ORR process. (**b**) Equivalent circuit

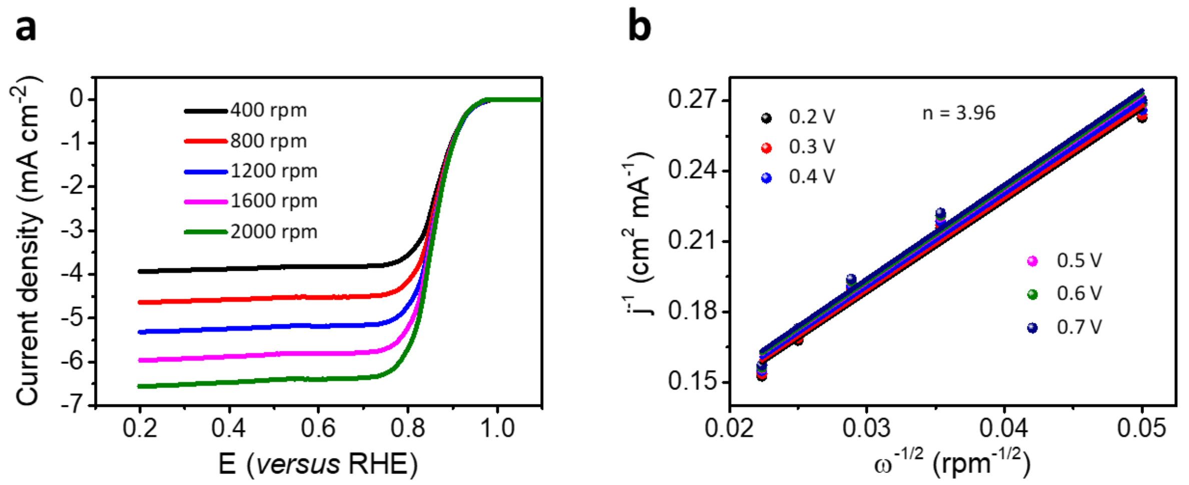

Fig. S6 Electrochemical kinetics. (**a**) LSV polarizations for Pt/C during ORR from 400 to 2000 rpm sweeping rates. (**b**) Determined K-L plots for 0.2 to 0.7 V

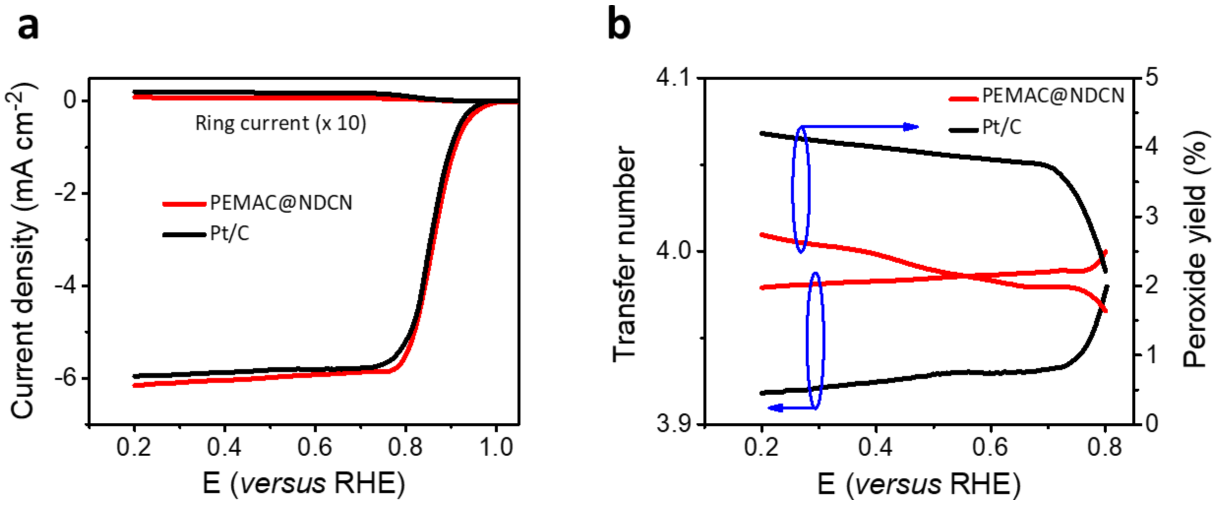

Fig. S7 Electrochemical kinetics. (**a**) Ring and disc current polarizations for PEMAC@NDCN and reference Pt/C catalysts. (**b**) Calculated electron transfer numbers and peroxide yield for PEMAC@NDCN and reference Pt/C

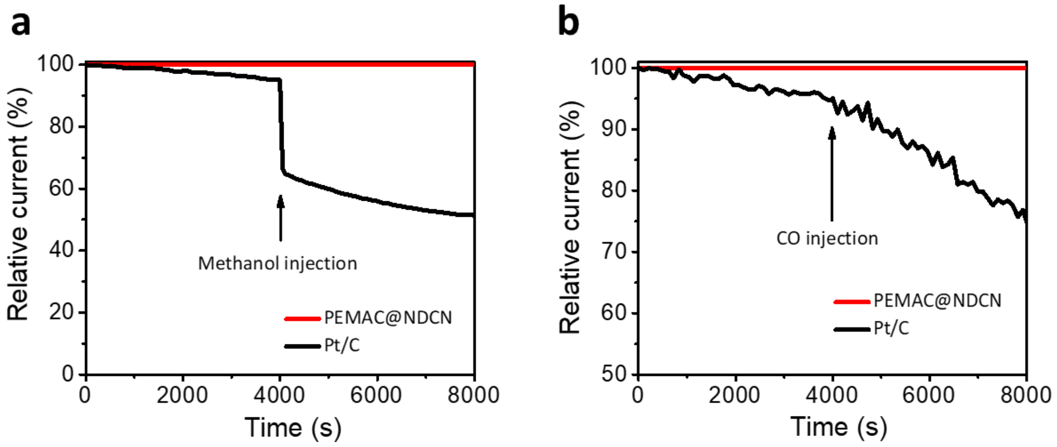

Fig. S8 Electrochemical performance. (**a**) Chronoamperometric I-t stability tests against methanol (2 M) resistance for PEMAC@NDCN and Pt/C. (**b**) Chronoamperometric I-t stability tests against CO (10 volume %) resistance for PEMAC@NDCN and Pt/C

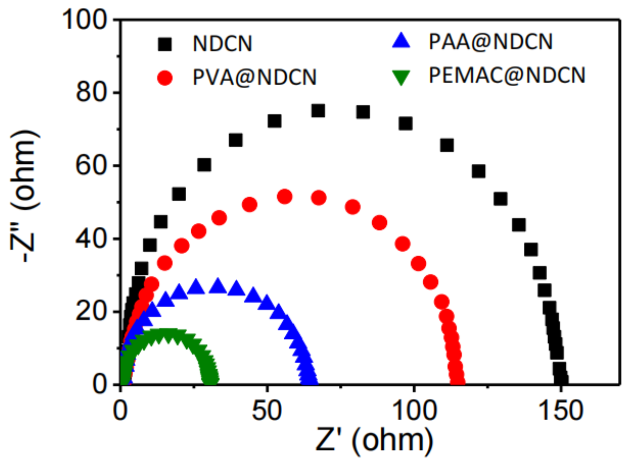

Fig. S9 Electrochemical kinetics for OER. EIS spectra for PEMAC@NDCN, PVA@NDCN, PAA@NDCN, and NDCN materials

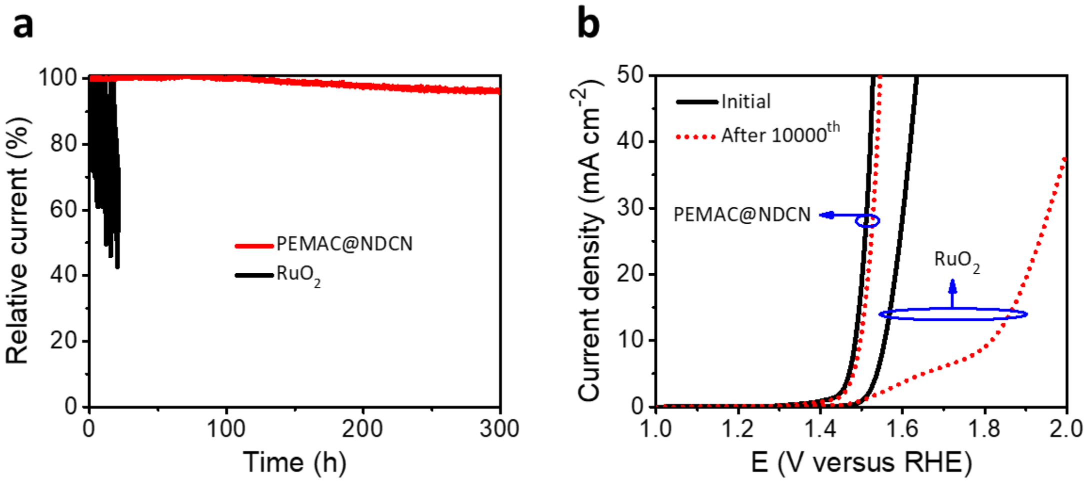

Fig. S10 Electrochemical stability testing for OER. (**a**) Chronopotentiometric polarizations obtained for PEMAC@NDCN compared to those of commercial RuO_2 at the respective overpotential. (**b**) LSV profiles for cycling operations (initial and after 10000th) for PEMAC@NDCN and RuO_2

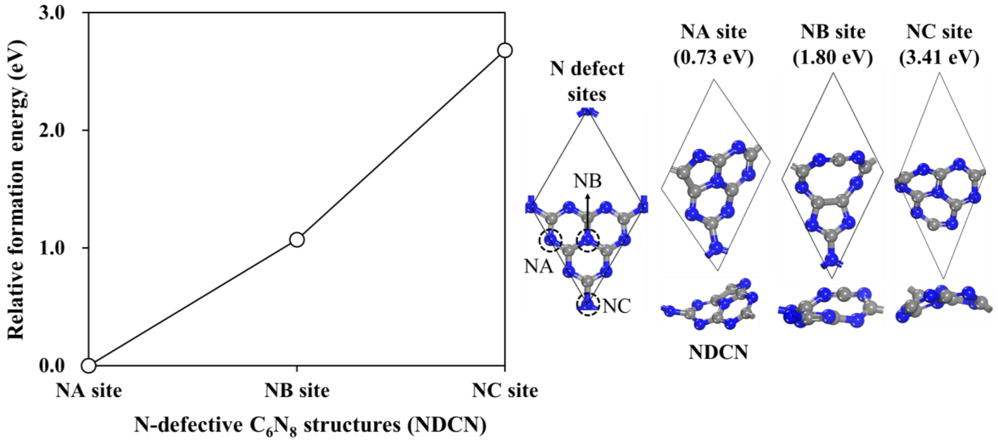

Fig. S11 Possible single N-defective C_6N_8 structures as N deficient C_3N_4 structure considering three different corner (NA), center (NB), and bridge (NC) N defect sites in C_3N_4 structure and formation energies, E_f, of them. NA-defective C_6N_8 structure is denoted as "NDCN"

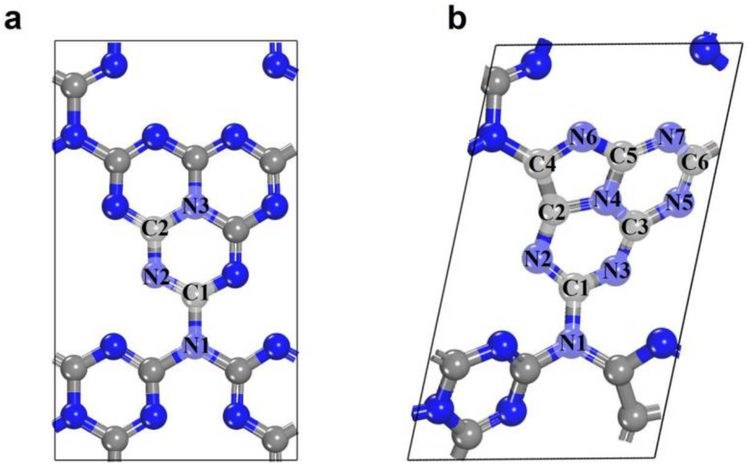

Fig. S12 Optimized structures of (**a**) Bulk CN and (**b**) NDCN. The insets represent active sites with element symbols and Arabic numbers

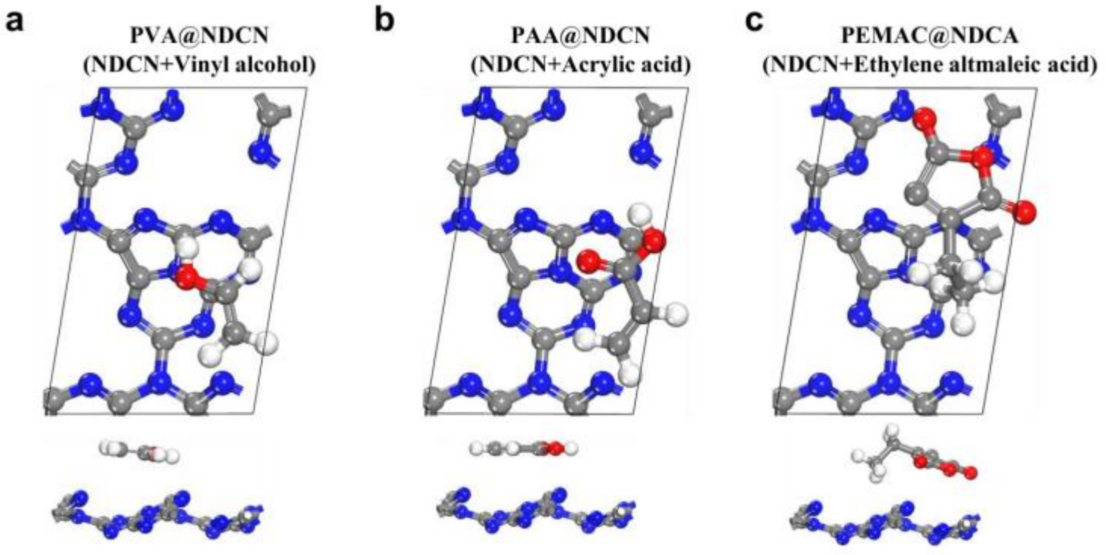

Fig. S13 Polymer-assisted NDCN model structures of (**a**) PVA@NDCN, (**b**) PAA@NDCN, and PEMAC@NDCN using polymer units

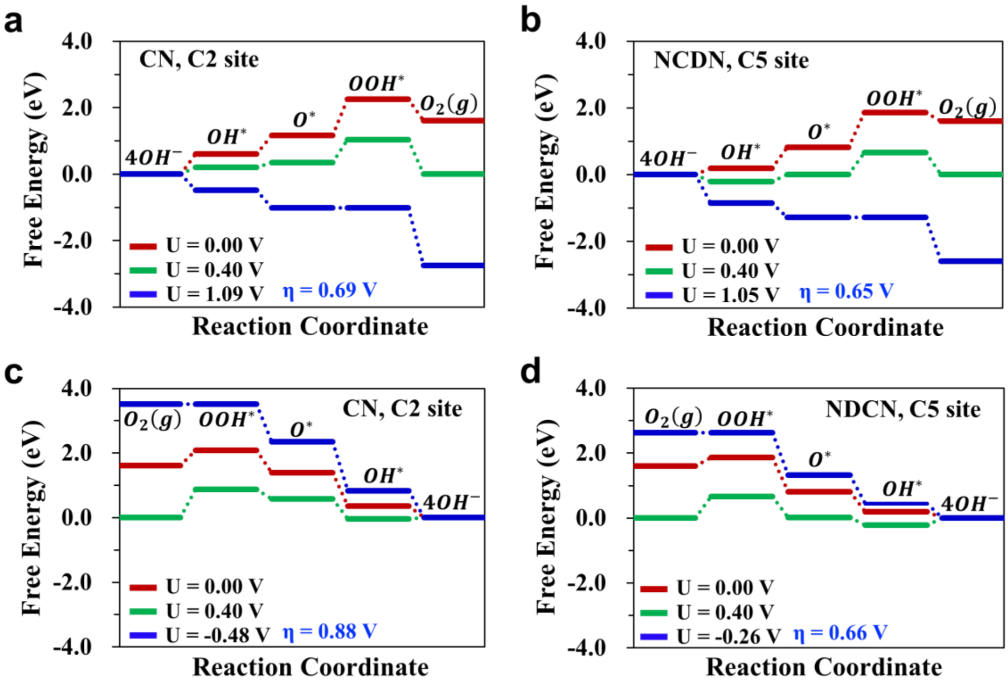

Fig. S14 (**a, b**) OER and (**c, d**) ORR free energy diagrams on bulk CN and NDCN in an alkaline environment

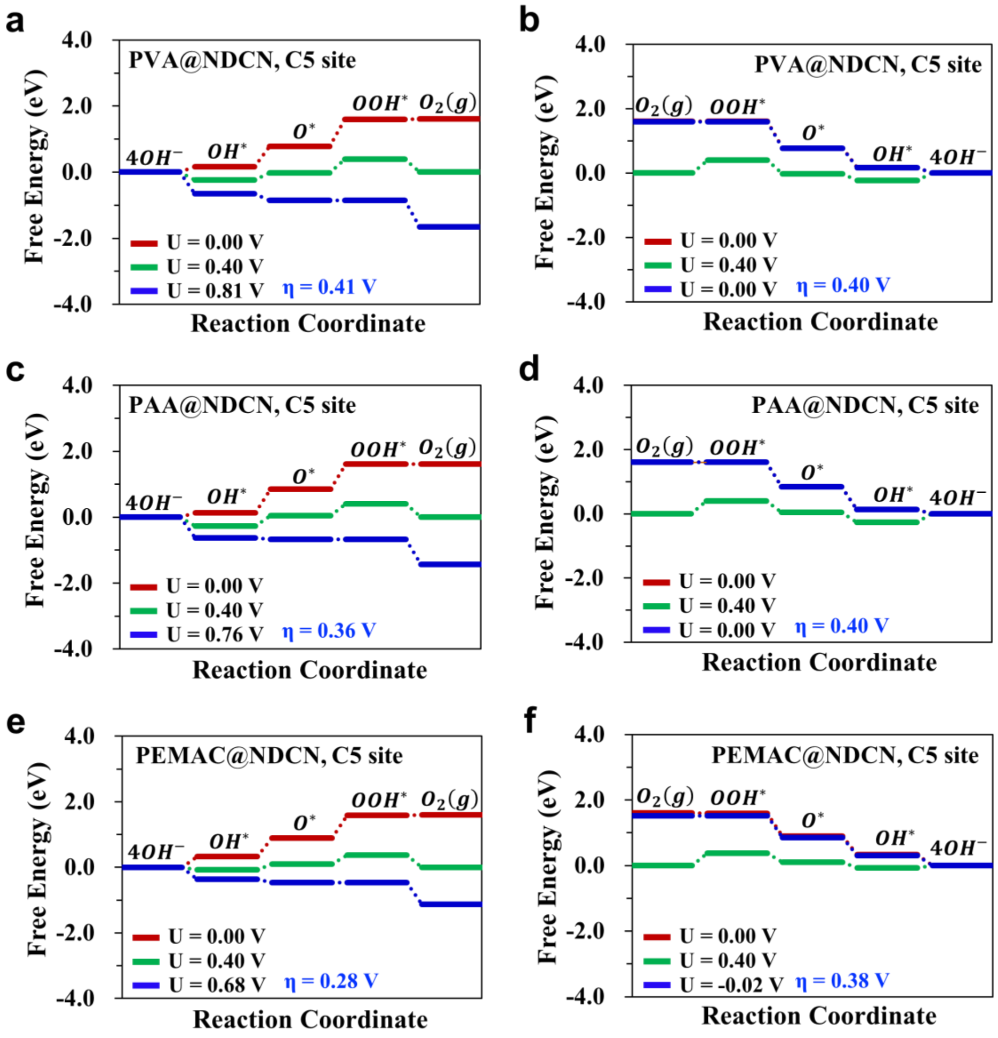

Fig. S15 OER and ORR free energy diagrams on (**a, b**) PVA@NDCN, (**c, d**) PAA@NDCN, and (**e, f**) PEMAC@NDCN in an alkaline environment

We performed a detailed analysis of the OER/ORR overpotentials ($\eta^{OER/ORR}$) by constructing free energy diagrams (FEDs) under different electrode potentials (U=0 V, 0.402 V and η V) (Supporting Information Equation S6-S21). From the FEDs for bulk CN, NDCN and polymer-assisted NDCN structures in Figs. S14 and S15, we can deduce the $\eta^{OER/ORR}$ corresponding to the largest endothermic reaction Gibbs free energy change (ΔG) under the equilibrium potential (U=0.402 V in alkaline media), denoted by green lines in Figs. S14-S15. Applying an additional $\eta^{OER/ORR}$ to overcome the potential-determining step ($G^{OER/ORR}$), we can finally get an overall downhill reaction represented by blue lines in FEDs. The calculated results reveal that the PEMAC@NDCN has the lowest overpotentials ($\eta^{OER/ORR}$) of 0.28 and 0.38 V, respectively. In addition, these values represent much better catalytic activities for conventional Pt (0.45 V) and RuO_2 (0.42 V).

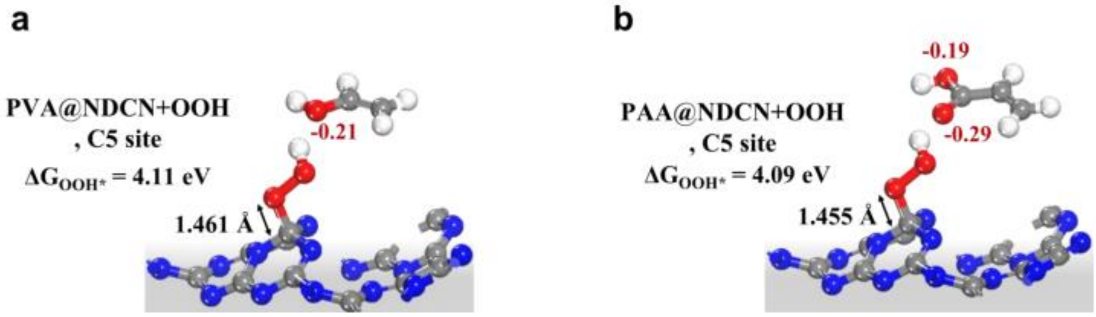

Fig. S16 The optimized structures of (**a**) PVA@NDCN and (**b**) PAA@NDCN with OOH* intermediate at the C5 active site

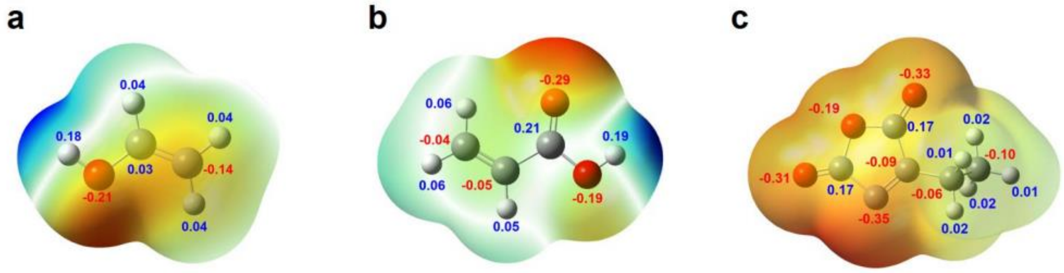

Fig. S17 Charge distribution of vinyl alcohol (VA), acrylic alcohol (AA) and ethylene-alt-maleic acid (EMA) monomers

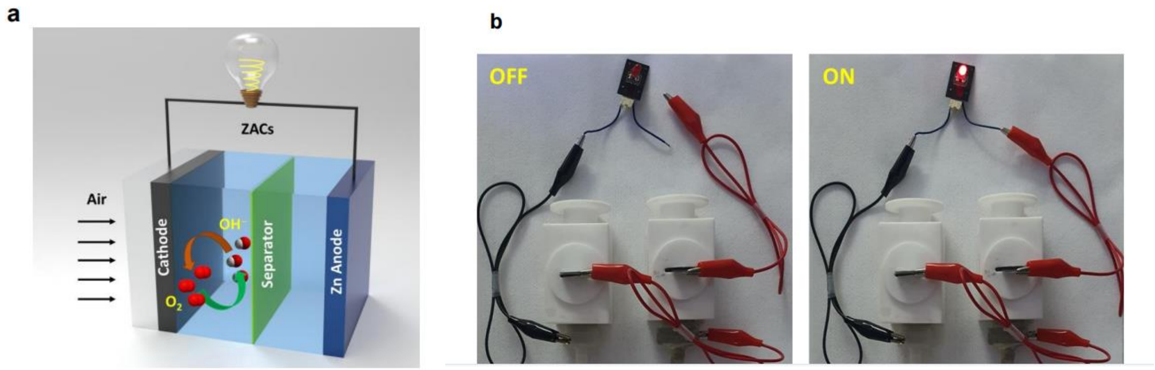

Fig. S18 Cell fabrication. (**a**) Design illustration for the rechargeable alkaline zinc air batteries. (**b**) Practical demonstration of assembled home-made liquid ZABs with red LED illumination

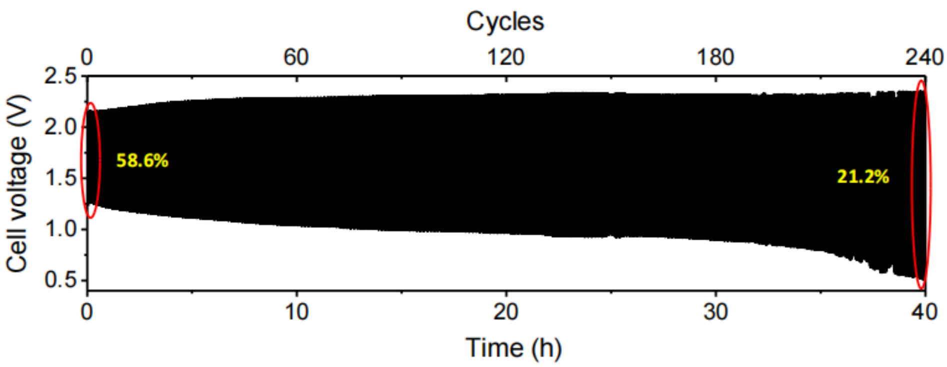

Fig. S19 Electrochemical performance for alkaline ZABs. Long-term cycle operations for discharge and charge states for Pt/C + RuO_2 based ZABs. (Conditions: Current density = 20 mA cm^{-2}, electrolyte = 6 M KOH + 0.2 M Zn acetate)

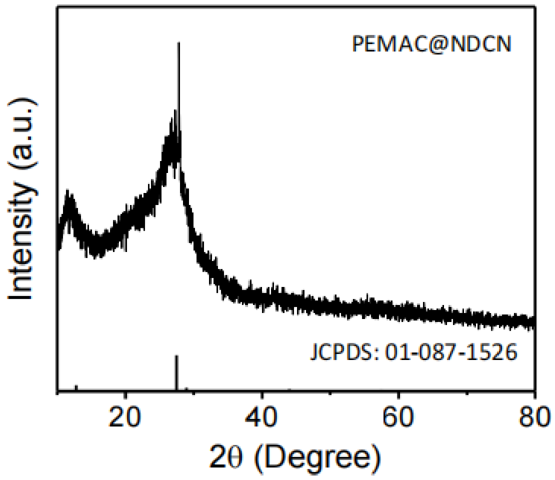

Fig. S20 Post spectroscopic characterizations of cathodes for alkaline ZABs. XRD patterns for PEMAC@NDCN over 2160 discharge and charge cycles. (see Fig. 8)

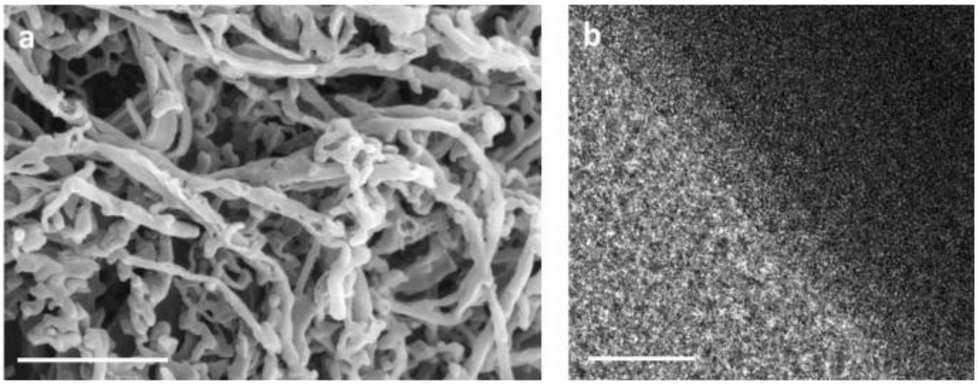

Fig. S21 Post spectroscopic characterizations of cathodes for alkaline ZABs. (**a**) SEM image for PEMAC@NDCN over 2100 discharge and charge cycles. (**b**) HRTEM image for PEMAC@NDCN over 2160 discharge and charge cycles. Scale 500 nm (**a**) and 3 nm (**b**). (see Fig. 8)

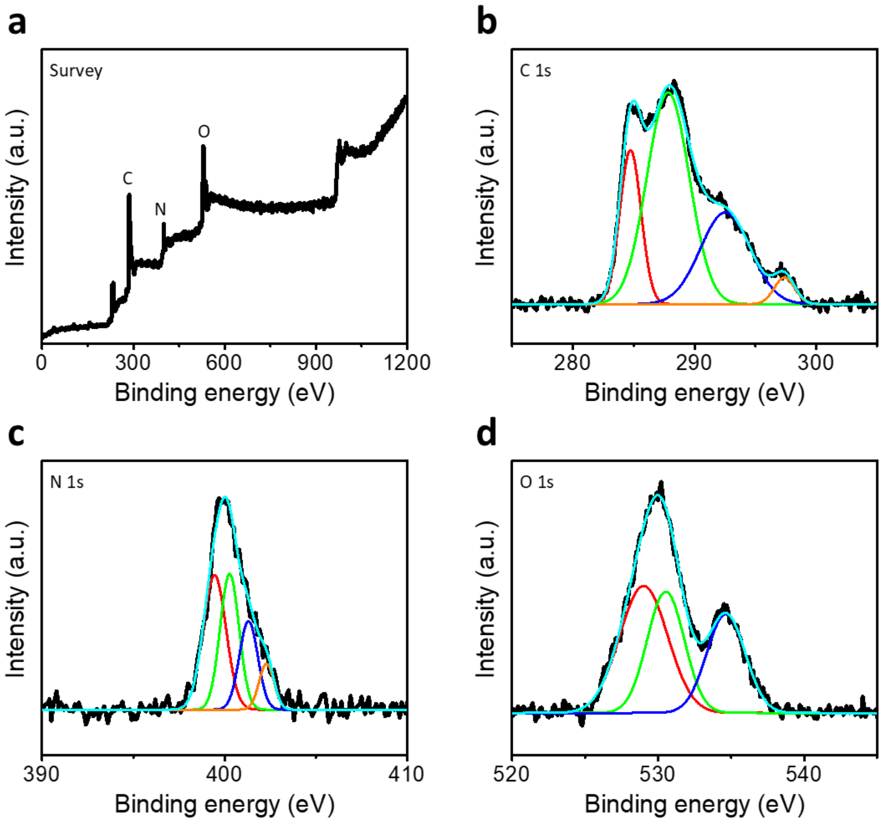

Fig. S22 Post spectroscopic characterizations of cathodes for alkaline ZABs. XPS spectra for PEMAC@NDCN over 2160 discharge and charge cycles. (**a**) Full scan, (**b**) C 1s, (**c**) N 1s, and (**d**) O 1s. (see Fig. 8)

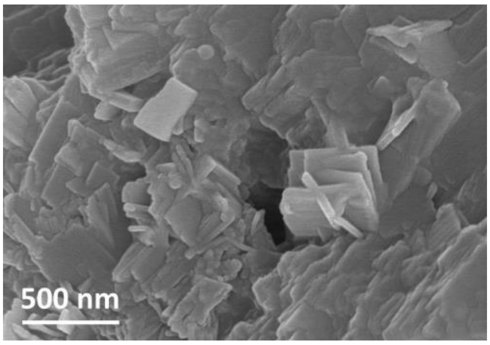

Fig. S23 Post microscopic characterization of Zn anode in alkaline ZABs. SEM image of utilized Zn anode after 2160 discharge and charge cycle operations. (see Fig. 8)

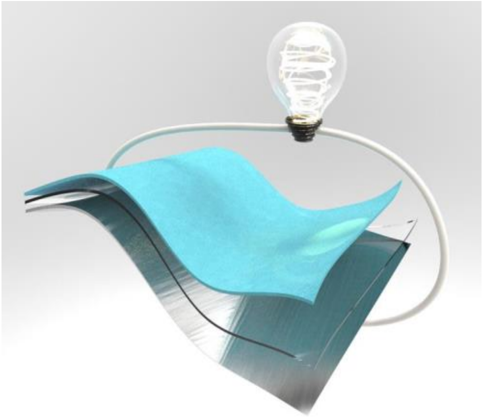

Fig. S24 Fabrication of flexible solid-state ZABs. Design illustration for the rechargeable flexible solid-state zinc air batteries

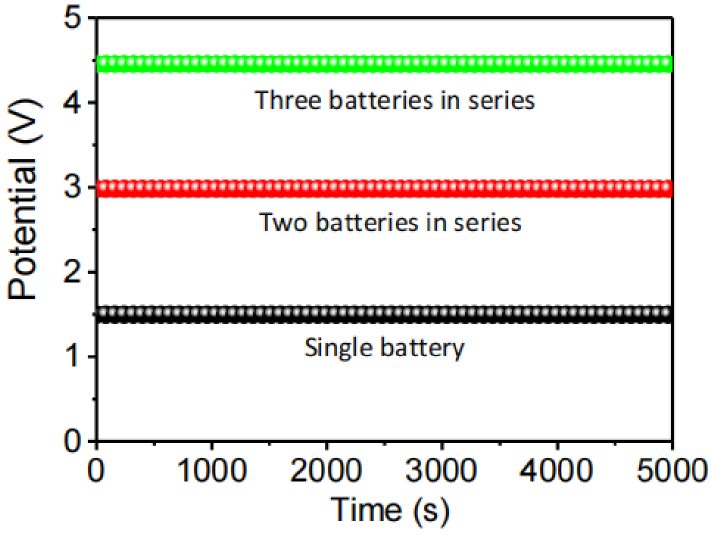

Fig. S25 Electrochemical performance of flexible ZABs. Comparison of open-circuit potentials (OCPs) over designed PEMAC@NDCN // CBCs // Zn cells for serial connections of one, two, and three cells

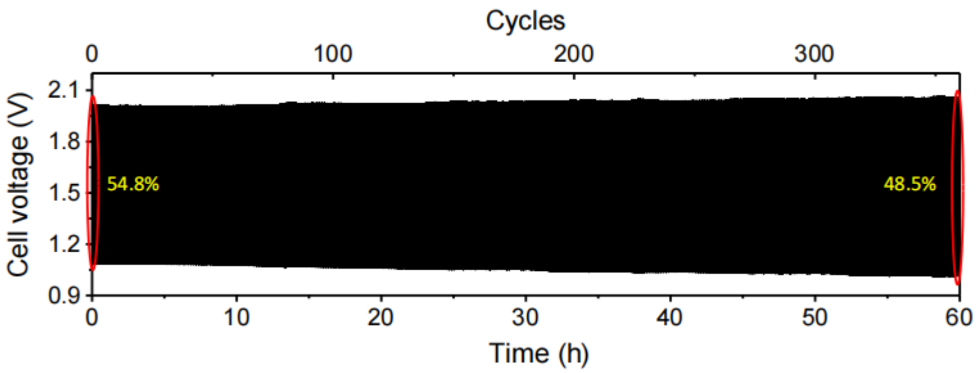

Fig. S26 Electrochemical performance for flexible ZABs. Cycle life assessments for Pt/C + RuO_2 // CBCs // Zn cells for the current rate of 50 mA cm^{-2} (Time: 10 min per cycle)

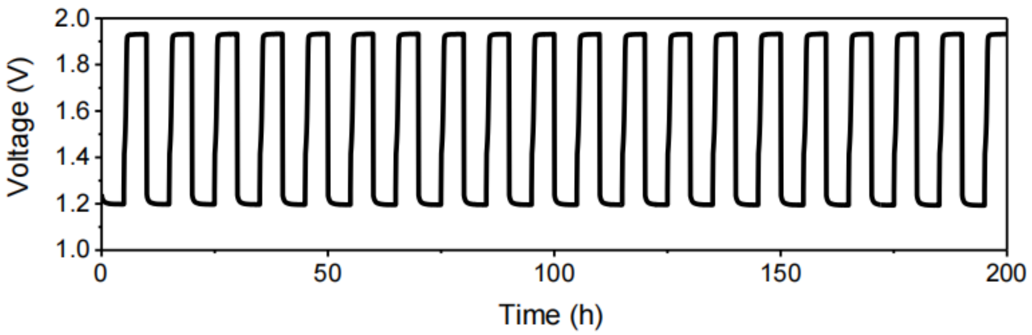

Fig. S27 Electrochemical performance for flexible ZABs. Discharge and charge cycle operations of PEMAC@NDCN // CBCs // Zn for 10 h per cycle at 50 mA cm^{-2}

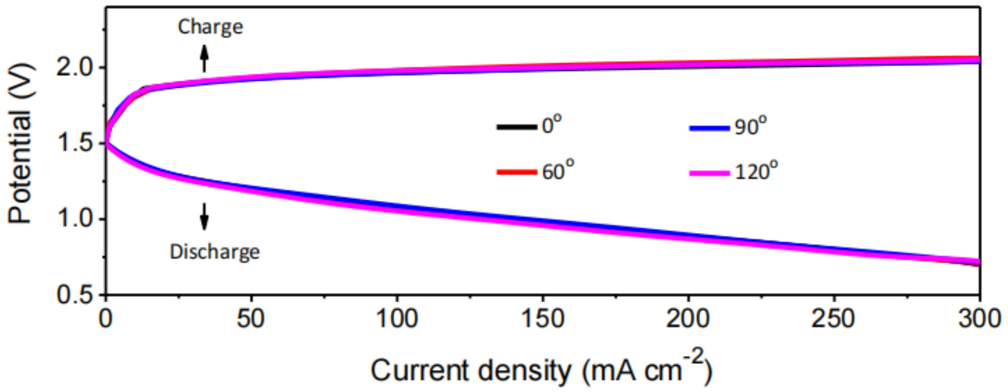

Fig. S28 Electrochemical performance for flexible ZABs. Discharge and charge mechanical bent testing (0°, 60°, 120°, and 180°) for PEMAC@NDCN // CBCs // Zn cells

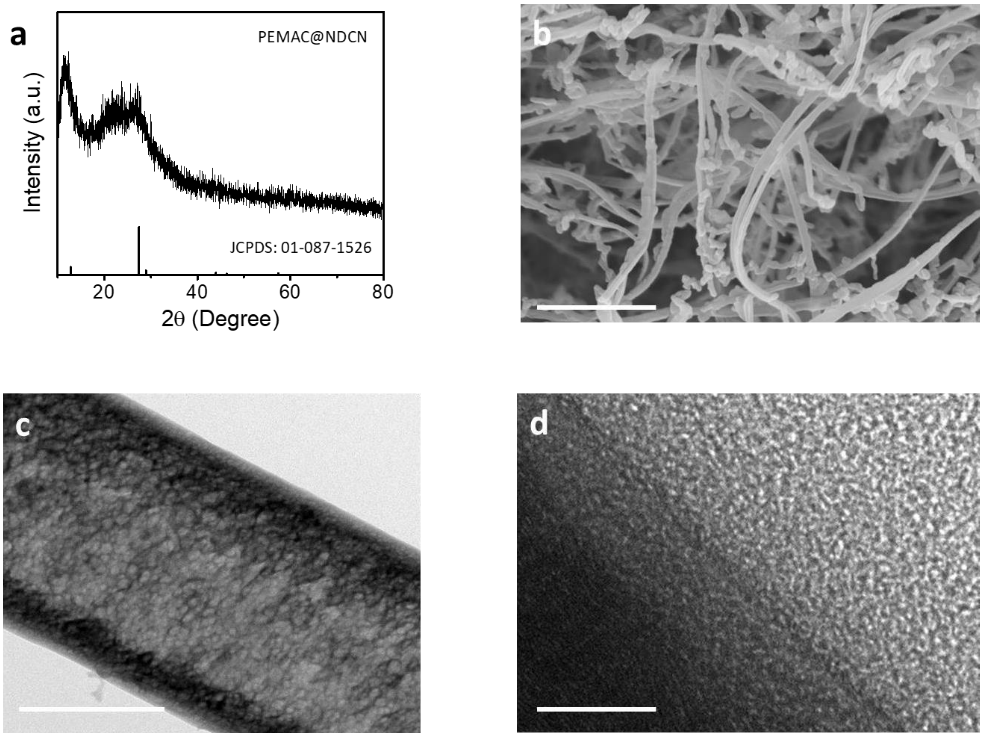

Fig. S29 Post spectroscopic characterizations of PEMAC@NDCN for flexible ZABs. (**a**) XRD pattern, (**b**) SEM image, (**c**) STEM image, and (**d**) HRTEM image for PEMAC@NDCN cathode after 2580 discharge-charge cycle operations (refer Fig. 9). Scale bars, 500 nm (**b**), 50 nm (**c**), and 3 nm (**d**)

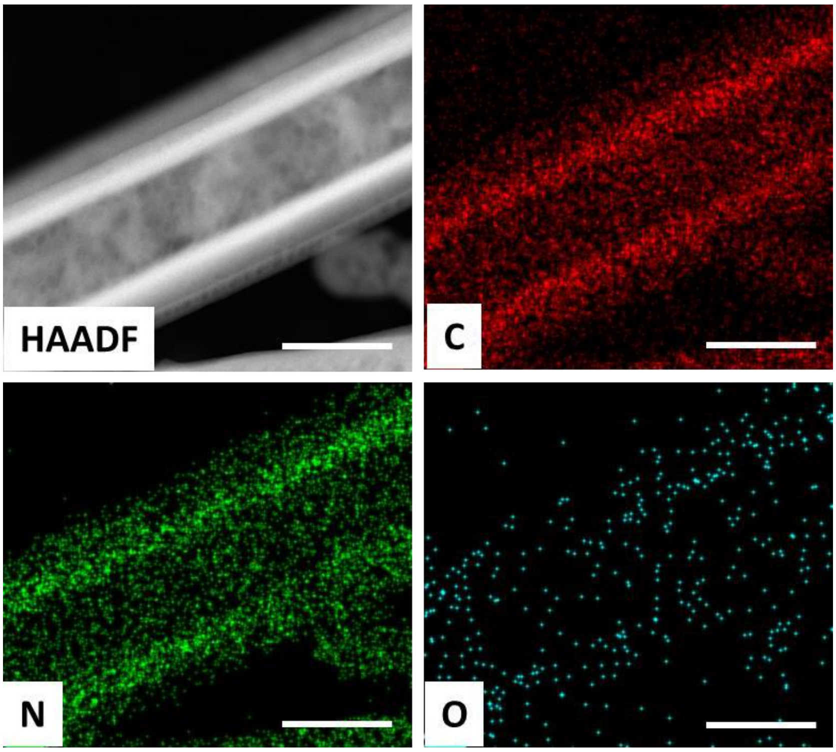

Fig. S30 Post elemental visualizations of PEMAC@NDCN for flexible ZABs. HAADF image and elemental maps for PEMAC@NDCN after 2580 discharge-charge cycle operations (refer to Fig. 9). Scale 50 nm (**a**)

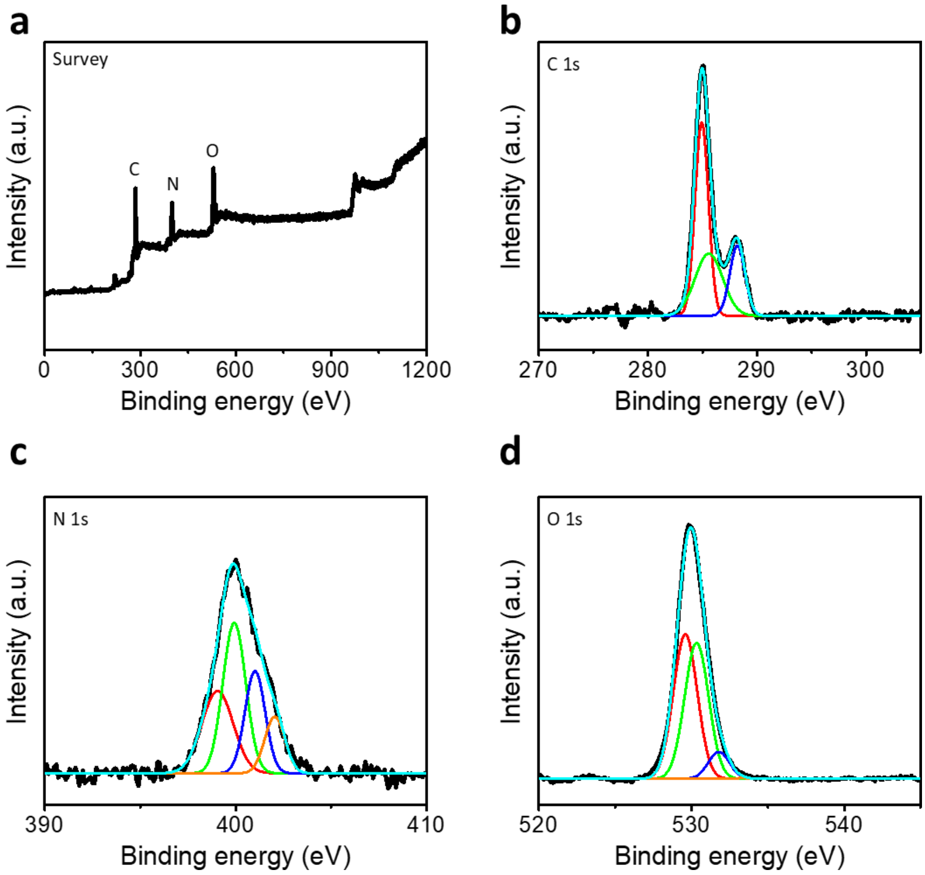

Fig. S31 Post spectroscopic characterizations of PEMAC@NDCN for flexible ZABs. XPS spectra for PEMAC@NDCN after 2580 discharge and charge cycle operations. (**a**) Full scan, (**b**) C 1s, (**c**) N 1s, and (**d**) O 1s (Fig. 9)

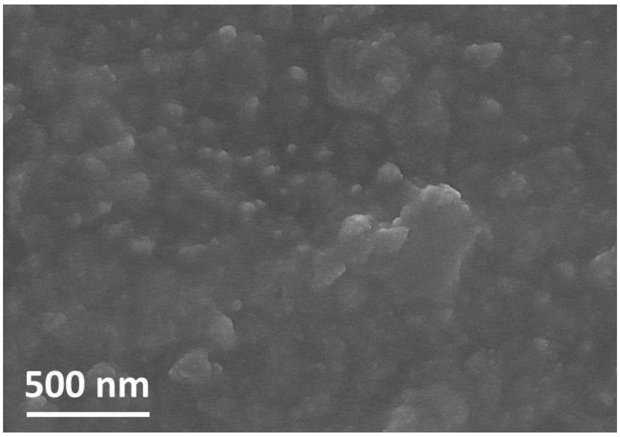

Fig. S32 Post microscopic characterization of Zn anode in flexible ZABs. SEM image of utilized Zn anode after 2580 discharge and charge cycle operations (Fig. 9)

S3 Supplementary Tables

Table S1 Quantitative elemental statistics for PVA@NDCN, PAA@NDCN, PEMAC@NDCN compared to NDCN and bulk CN catalysts using XPS and EDS characterizations

Sample	C (at%)[a]	N (at.%)[a]	O (at%)[a]	C (at%)[b]	N (at%)[b]	O (at%)[b]
Bulk CN	42.13	56.09	1.78	42.19	55.92	1.89
NDCN	52.68	44.23	3.09	52.83	44.01	3.16
PVA@NDCN	59.2	33.69	7.11	59.23	33.71	7.06
PAA@NDCN	58.78	34.05	7.17	58.75	33.99	7.26
PEMAC@NDCN	58.9	33.24	7.86	59.13	33.18	7.69

Note the "a" and "b" represents the XPS and EDS results

Table S2 Quantitate estimation of nitrogen species: pyridinic (N1), pyrrolic N (N2), graphitic (N3), quaternary (N4), and oxidized N (N5) for the fabricated polymeric catalysts

Sample	N1	N2	N3	N4	N5
Bulk CN	-	399.42	400.95	-	-
NDCN	-	399.38	400.12	401.52	-
PVA@NDCN	-	399.5	400.30	401.39	402.41
PAA@NDCN	-	399.41	400.12	401.17	402.40
PEMAC@NDCN	398.81	399.59	400.43	401.61	402.62

Table S3 Influence of polymer to N deficient carbon nitride ratio over electrochemical performances

**Polymer content (%)**	**Overall oxygen bifunctional activity E (V)**
0	0.93
1	0.91
3	0.79
5	0.61
7	0.66
10	0.86
20	1.06

Table S4 Comparison for bifunctional ORR and OER performance for the fabricated polymeric and previously reported champion catalysts

Catalyst	Loading (mg cm^{-2})	ORR onset potential (V *vs.* RHE)	ORR Tafel slope (mV dec^{-1})	ORR half-wave potential ($E_{1/2}$) (V *vs.* RHE)	OER onset potential (V *vs.* RHE)	OER Tafel slope (mV dec^{-1})	OER potential @ 10 mA cm^{-2} ($E_{j=10}$) (V *vs.* RHE)	Overall oxygen electrode activity E ($E_{j=10}$ - $E_{1/2}$) (V)	Refs.
PEMAC@NDCN	0.15	-	42	0.87	-	48	1.48	0.61	This work
Mn/Fe-HIB-MOF	0.15	0.98	36	0.883	1.33	45	1.51	0.63	[S10]
Fe/SNCFs-NH_3	-	-	70.82	0.89	-	-	1.78	0.89	[S11]
CPS(101)	0.1	0.99	39	0.90	1.38	58	1.49	0.59	[S12]
FeCo SAs@Co/N-GC	0.25	0.98	49	0.88	1.44	56.6	1.52	0.64	[S13]
Ir@Co_3O_4[a]	0.5	-	-	0.75	-	-	1.51	0.76	[S14]
Ni SAs-NC[a]	0.1	-	-	0.85	-	62	1.67	0.82	[S15]
Co-FPOH[a]	0.283	0.82	183	0.69	-	63	1.52	0.83	[S16]
Co@IC/MoC@PC	0.4	-	78	0.875	-	82	1.51	0.635	[S17]
Nitride/N-Ti_3C_2	1.0	0.95	-	0.84	1.48	72	1.53	0.69	[S18]
IOSHs-NSC-Co_9S_8	1	-	-	0.82	-	101	1.64	0.82	[S19]
NPMC-1000	0.10	0.94	-	0.85	1.30	-	-	-	[S20]
NiO/CoN PINWs	0.20	0.89	35	0.68	1.53	-	1.53	0.85	[S21]
PS-CNS	0.15	0.97	61	0.87	1.26	64	1.56	0.69	[S22]
Co_4N/CNW/CC	0.20	0.89	-	0.80	1.50	81	1.54	0.74	[S23]
CCO@C	0.2	0.95	-	0.86	1.47	74	1.56	0.70	[S24]
1100-CNS	0.42	0.99	58	0.88	1.50	292	1.69	0.81	[S25]

C-MOF-C2-900	0.5	0.92	-	0.817	1.50	79	1.58	0.763	[S26]
Co-N,B-CSs	0.1	0.89	64	0.83	1.42	-	1.66	0.83	[S27]
S-C_2NA	0.15	0.98	54	0.88	1.28	62	1.53	0.65	[S28]
FeN_x-PNC	0.14	0.99	-	0.86	1.56	80	1.63	0.77	[S29]
CFZr(0.3)/N-rGO	0.3	0.85	-	0.76	1.4	-	1.6	0.84	[S30]
NDGs-800	0.2	0.98	81	0.85	1.5	132	1.68	0.83	[S31]
CMO/S-300	0.3	0.915	52	0.76	1.5	-	1.70	0.94	[S32]
Co/Co_3O_4@PGS	0.3	0.97	52.6	0.89	1.52	76.1	1.58	0.69	[S33]
DN-CP@G	0.2	0.95	-	0.801	1.5	65	1.788	0.987	[S34]
N-GRW	0.30	0.92	53	0.84	1.53	47	1.59	0.75	[S35]
NCNF-1000	0.10	0.94		0.85	1.30		1.84	1.02	[S36]
$NiCo_2S_4$/N-CNT	0.25	0.93	-	0.80	-	-	1.60	0.80	[S37]
NC-Co_3O_4-90	1.2	0.91	-	0.87	-	-	1.59	0.72	[S38]
FeNi@N-GR	0.2	0.94	-	0.83	1.38	62	1.44	0.61	[S39]
PS-CNF	0.2	0.95	29	0.86	1.32	89	1.55	0.69	[S40]
S,N-Fe/N/C-CNT	0.25	0.94	-	0.84	1.45	82	1.60	0.76	[S41]
Co_3O_4/NPGC	0.20	0.97	-	0.84	1.45	-	1.68	0.84	[S42]
PCN-CFP	0.20	0.94	122.3	0.67	1.53	61.6	1.63	0.96	[S43]
N-GCNT/FeCo-3	0.40	1.03	66.8	0.92	1.42	99.5	1.73	0.81	[S44]
CoO/N-G	0.70	0.90	48	0.81	1.30	71	1.57	0.76	[S45]
S-GNS/$NiCo_2S_4$	0.3	0.93	-	0.88	1.48	65	1.56	0.69	[S46]

][

Table S5 Comparison over electrochemical performances of primary ZABs under in alkaline electrolytes for previously reported champion materials

Catalyst	OCP (V)	Power density (mW cm^{-2})	Specific capacity (mAh g^{-1})	Energy density (Wh kg^{-1})	Durability (h)	Refs.
PEMAC@NDCN	1.51	214	817	1070	-	This work
Pt/C +RuO_2	1.5	178	773	966	-	This work
Mn/Fe-HIB-MOFs	1.50	195	768	1027	-	[S10]
Fe/SNCFs-NH_3	-	255	-	-	-	[S11]
FeCo SAs@Co/N-GC	-	207	741	934	-	[S13]
Co-FPOH	-	167.8	817	980	-	[S16]
Co@IC/MoC@PC	-	221	728	-	-	[S17]
Nitride/N-Ti_3C_2	-	27	630	756	-	[S18]
PFN PF	-	175	816	938	-	[S47]
Co_9S_8@N,S-C	-	259	862	-	-	[S48]
FeCo/Se-CNT	-	175	750	894	-	[S49]
Co-TMPyP/CCG	-	225	793	-	-	[S50]
NPMC-1000	1.48	55	735	-	240 (~1.3 V)	[S20]
NiO/CoN PINWs	1.46	79.6	690	945	-	[S21]
P,S-CNS	1.51	198	830	-	210 (~1.3 V)	[S22]

Table S6 Comparison over cycle life assessment of rechargeable ZABs in the alkaline electrolyte

Catalyst	Rechargeability	Refs.
PEMAC@NDCN	600 s/cycle for 2160 cycles; 360 h@20 mA cm^{-2}	This work
Mn/Fe-HIB-MOFs	600 s/cycle for 6000 cycles; 1000 h@10 mA cm^{-2}	[S10]
Fe/SNCFs-NH_3	3600 s/cycle for 1000 cycles; 1000 h@1 mA cm^{-2}	[S11]
FeCo SAs@Co/N-GC	600 s/cycle for 1200 cycles; 200 h@10 mA cm^{-2}	[S13]
Ir@Co_3O_4	1200 s/cycle for 720 cycles; 240 h@10 mA cm^{-2}	[S14]
Co-FPOH	600 s/cycle for 1200 cycles; 450 h@5 mA cm^{-2}	[S16]
Co@IC/MoC@PC	1200 s/cycle for 300 cycles; 100 h@1 mA cm^{-2}	[S17]
Nitride/N-Ti_3C_2	3600 s/cycle for 120 cycles; 120 h@20 mA cm^{-2}	[S18]
PFN PF	1800 s/cycle for 1000 cycles; 500 h@20 mA cm^{-2}	[S47]
Co_9S_8@N,S-C	720 s/cycle for 660 cycles; 110 h@1 mA cm^{-2}	[S48]
FeCo/Se-CNT	1200 s/cycle for 210 cycles; 70 h@5 mA cm^{-2}	[S49]
Co-TMPyP/CCG	1200 s/cycle for 300 cycles; 100 h@2 mA cm^{-2}	[S50]
NPMC-1000	600 s/cycle for 600 cycles; 100 h@2 mA cm^{-2}	[S20]
NiO/CoN PINWs	600 s/cycle for 25 cycles; 8.3 h@50 mA cm^{-2}	[S21]
P,S-CNS	720 s/cycle for 500 cycles; 100 h@2 mA cm^{-2}	[S22]
Co_4N/CNW/CC	1200 s/cycle for 408 cycles; 136 h@10 mA cm^{-2}	[S23]
CCO@C	1800 s/cycle for 160 cycles; 80 h @2 mA cm^{-2}	[S24]
1100-CNS	660 s/cycle for 300 cycles; 55 h @10 mA cm^{-2}	[S25]
C-MOF-C2-900	1200 s/cycle for 360 cycles; 120 h @2 mA cm^{-2}	[S26]

Co-N,B-CSs	394 s/cycle for 128 cycles; 14 h @5 mA cm^{-2}	[S27]
S-C_2NA	2 h/cycle for 375 cycles; 750 h@10 mA cm^{-2}	[S28]
FeN_x-PNC	660 s/cycle for 220 cycles; 40 h @5 mA cm^{-2}	[S29]
CFZr(0.3)/N-rGO	3600 s/cycle for 11 cycles; 11 h @15 mA cm^{-2}	[S30]
NDGs-800	1200 s/cycle for 234 cycles; 78 h @10 mA cm^{-2}	[S31]
CMO/S-300	400 s/cycle for 120 cycles; 13 h @5 mA cm^{-2}	[S32]
Co/Co_3O_4@PGS	600 s/cycle for 4800 cycles; 800 h @10 mA cm^{-2}	[S33]
DN-CP@G	3600 s/cycle for 250 cycles; 250 h @5 mA cm^{-2}	[S34]
N-GRW	1h/cycle for 160 cycles; 160 h@2 mA cm^{-2}	[S35]
NCNF-1000	600 s/cycle for 500 cycles; 83 h@10 mA cm^{-2}	[S36]
$NiCo_2S_4$/N-CNT	400 s/cycle for 150 cycles; 17 h@10 mA cm^{-2}	[S37]
NC-Co_3O_4-90	1200 s/cycle for 600 cycles; 200 h@10 mA cm^{-2}	[S38]
FeNi@N-GR	1200 s/cycle for 120 cycles; 40 h @20 mA cm^{-2}	[S39]
PS-CNF	700 s/cycle for 600 cycles; 120 h @2 mA cm^{-2}	[S40]
Co_3O_4/NPGC	1200 s/cycle for 250 cycles; 83 h@5 mA cm^{-2}	[S42]
PCN-CFP	600 s/cycle for 50 cycles; 8.3 h@20 mA cm^{-2}	[S43]
N-GCNT/FeCo-3	600 s/cycle for 240 cycles; 40 h@125 mA cm^{-2}	[S44]
S-GNS/$NiCo_2S_4$	2400 s/cycle for 150 cycles; 100 h @10 mA cm^{-2}	[S51]
CNT@POF	1200 s/cycle for 200 cycles; 67 h @2 mA cm^{-2}	[S52]
CoO/N-CNT + NiFe LDH/Ni	10 h/cycle for 10 cycles; 200 h@20 mA cm^{-2}	[S57]
CuS/NiS_2	1800 s/cycle for 500 cycles; 83 h @25 mA cm^{-2}	[S60]
macro/meso-NC-NH_3 + COMT@Ni	4 h/cycle for 200 cycles; 800 h@10 mA cm^{-2}	[S61]
Co_3O_4 NWs	600 s/cycle for 100 cycles; 17 h@50 mA cm^{-2}	[S62]
NCNT/CoO-NiO-NiCo	600 s/cycle for 100 cycles; 17 h@20 mA cm^{-2}	[S63]
LBSCFO-50	600 s/cycle for 100 cycles; 17 h@10 mA cm^{-2}	[S64]
$Ni_{6/7}Fe_{1/7}$-OH-6/CNT	600 s/cycle for 150 cycles; 25 h@15 mA cm^{-2}	[S65]
NiCo/PFC	2 h/cycle for 300 cycles; 600 h@10 mA cm^{-2}	[S66]
Ni_3Fe/N-C	4 h/cycle for 105 cycles; 420 h@10 mA cm^{-2}	[S67]
NiO/$Ni(OH)_2$	70 min/cycle for 70 cycles; 82 h@1 mA cm^{-2}	[S68]
Fe@NC	600 s/cycle for 100 cycles; 17 h@10 mA cm^{-2}	[S69]
BNC	600 s/cycle for 66 cycles; 11 h@20 mA cm^{-2}	[S70]
c-$CoMn_2$/C	400 s/cycle for 155 cycles; 17 h@10 mA cm^{-2}	[S71]
CCBC-2	600 s/cycle for 75 cycles; 12.5 h@2 mA cm^{-2}	[S72]
$RuSn_{73}$	600 s/cycle for 100 cycles; 16.6 h@10 mA cm^{-2}	[S73]
α-MnO_2/CNT_{10}	300 s/cycle for 100 cycles; 8.33 h@10 mA cm^{-2}	[S74]

Table S7 Comparison over electrochemical performances of flexible ZABs with previously reported champion catalysts and electrolytes.

Catalyst	Electrolyte	Power density (mW cm^{-2})	Specific capacity (mAh g_{Zn}^{-1})	Energy density (Wh kg_{Zn}^{-1})	Recharge ability	Refs.
PEMAC@NDCN	CBCs	211	806	1056	10 min/cycle for 2580 cycles; 430 h@50	This work
Mn/Fe-HIB-MOF	Functionalized Bio-cellulose membrane	193	748	975	10 min/cycle for 3600 cycles; 600h@25	[S10]
FeCo SAs@Co/N-GC	Functionalized Bio-cellulose	186	817	1017	10 min/cycle for 684 cycles; 114 h@50	[S13]
CPS(101)	CBCs	458	-	-	8 min/cycle for 6000 cycles; 800 h@25	[S12]
Fe/SNCFs-NH3	(PANa)-KOH-$Zn(CH_3COO)_2$ hydrogel	-	-	-	30 min/cycle for 120 cycles; 60 h@1	[S11]
FeCo/Se-CNT	PVA gel	37.5	-	-	10 min/cycle for 120 cycles; 20 h@5	[S49]
Fe1/d-CN	PVA gel	78	-	-	20 min/cycle for 60	[S75]
Nitride/N-Ti_3C_2	PVA gel	27	630	693	60 min/cycle for 120 cycles; 120 h@20	[S18]
CuSA@HNCNx	Functionalized Bio-cellulose	212	806	1031	10 min/cycle for 1500 cycles; 250 h@ 25	[S76]
Co4N/CNW/CC	PVA gel	-	-	-	20 min/cycle for 36 cycles; 12 h	[S23]
Co-N,B-CSs	PVA gel	-	-	-	6 min/cycle for 230 cycles; 23 h@2	[S27]
S-C_2NA	Functionalized Cellulose film	187	695	862	30 min/cycle for 900 cycles; 450 h	[S28]
CMO/S-300	PVA gel	-	-	-	30 min/cycle for 20 cycles; 10 h@1	[S32]
DN-CP@G	PVA gel	-	530	-	60 min/cycle for 170 cycles; 170 h@1	[S34]
NCNF-1000	PVA gel	-	378	378	10 min/cycle for 48 cycles; 8 h@1	[S36]
NC-Co_3O_4-90	Acrylic acid gel	82	387.2	387.2	24 min/cycle for 60 cycles; 24 h@1	[S38]
FeNi@N-GR	PVA gel	-	-	-	20 min/cycle for 54 cycles; 18 h	[S39]
N-GCNT/FeCO-3	PVA gel	97.8	-	-	10 min/cycle for 72 cycles; 12 h	[S44]
CNT@POF	PVA gel	-	-	-	4 min/cycle for 12 cycles; 48 min	[S52]
CuS/NiS_2	PVA gel	-	-	-	10 min/cycle for 150 cycles; 25 h	[S60]
Co_3O4-NCNT/SS	Cellulose film	162	652.6	847.6	20 min/cycle for 1500 cycles; 500 h@25	[S77]
MnO_x-GCC	Acrylic acid gel	32	-	-	20 min/cycle for 200 cycles; 66.66 h@0.7	[S78]
Co_3O_4	Cellulose membrane	-	-	-	1 h/cycle for 35 cycles; 35 h@250	[S79]
$LaNiO_3$/NCNT	PVA gel	56.1	460	581	20 min/cycle for 120 cycles; 40 h@50	[S80]

Table S8 Comparison of flexible ZABs performances over wide temperature with previously champion reports

Catalyst	Electrolyte	Lowest working temperature (°C)	Working temperature (°C)	Power density (mW cm^{-2})	Specific capacity (mAh g_{Zn}^{-1})	Energy density (Wh kg_{Zn}^{-1})	Rechargeability @mA cm^{-2}	Refs.
PEMAC@NDCN	CBCs	-40	70<br>-40	224<br>159	817<br>693 (86%)	1074<br>738 (~70%)	580 cycles (96 h) @25<br>550 cycles (91 h) @25	This work
CPS(101)	CBCs	-20	80<br>-20	458<br>-	1.24 Ah<br>1 Ah	-<br>-	1500 cycles (200 h) @25<br>1500 cycles (200 h) @25	Nat. Energy **6,** 592-604 (2021)
FeMn-DSAC	PAM	-40	25<br>-40	184<br>30	734<br>631 (86%)	888<br>725 (81.6%)	218 cycles (80 h) @2<br>218 cycles (80 h) @2	Angew. Chem. Int. Ed. **61,** e202115219 (2022)
$Ni_{0.8}Fe_{0.2}$ NSs	PVA	-10	25<br>-10	-<br>-	-<br>-	-<br>-	15 h @1<br>15 h @1	ChemCatChem **11**, 6002 (2019)
NiO/CoO TINWs	PVA	-10	25<br>-10	-<br>-	842<br>328 (39%)	996<br>364 (36%)	33 h @1<br>14 h @1	Angew. Chem. Int. Ed. **58,** 9459 (2019)
Co@NPCP/NBC NF- 2-800	PAM	-50	25<br>-50	165<br>97	800<br>620 (77%)	960	600 cycles (100 h) @2<br>240 cycles (40 h) @2	Energy Environ. Sci. **14,** 4451 (2021)
$NiCo_{2.148}O_4$ PNSs	PVA	-35	25<br>-35	83<br>-	-<br>-	910	1200 cycles (20 h) @3	Adv. Mater. **32**, 2001651 (2020)
BFC-FC-0.2	PAA	-20	25<br>-20	160<br>80	745<br>691 (92%)	915<br>798 (87%)	600 cycles (105 h) @2<br>-	Angew. Chem. Int. Ed. **59,** 4793 (2020)
Pt/C + Ir/C	KOH<br>CsOH	-20	20<br>-20	126<br>43	-<br>-	-<br>-	210 cycles @5<br>255 cycles @5	Angew. Chem. Int. Ed. **60,** 15281 (2021)
Pt/RuO_2	PAM	-40	20<br>-40	43<br>10	818<br>743 (90%)	-<br>-	75 h @2<br>40 h @2	Energy Stor. Mater. **42,** 88 (2021)
Pt/RuO_2	PANa	-20	25<br>-20	60<br>21	673<br>-	-<br>-	86 cycles (29 h) @1<br>133 cycles (44 h) @1	Compos. B. Eng. 109228 (2021)
Pt/C and RuO_2	PAM/PAA	-20	25<br>-20	12<br>8	663<br>506 (76%)	769<br>556 (72%)	100 cycles (10 h) @1<br>100 cycles (10 h) @1	ACS Sustain. Chem. Eng. **8,** 11501 (2020)
Co_3O_4	PAM	-20	25<br>-20	73<br>52	764<br>-	850<br>-	70 cycles (24 h) @1<br>-	ACS Appl. Mater. Interfaces **12,** 11778 (2020)

S4 Note S2: Computational Details and Modeling

All *ab initio* calculations were performed with the Vienna *Ab initio* Simulation Package (VASP 5.4.4) [S81-S84]. We used the BEEF-vdW [S85] exchange-correlation functional using the projector augmented wave (PAW) method [S86, S87] with a generalized gradient to accurately describe the chemisorption as well as physisorption on the catalyst surface. Integration in the Brillouin zone was performed based on the Monkhorst-Pack scheme using a Γ-centered 3 × 2 × 1 k-point mesh in each primitive lattice vector of the reciprocal space for geometric optimization. Plane-wave cutoff energy of 500 eV was used. Lattice constants and internal atomic positions were fully optimized until the residual forces were less than 0.04 eV $Å^{-1}$. The vacuum slab space of a unit cell in the z-direction was set to 16 Å to avoid interactions between layers. The schematics of our models are shown as Figs. S12-S13. For the convenience of identifying the active site positions, we name them by element name and Arabic number, and the details of the active site naming are included in Figs. 7a and S12.

S4.1 OER and ORR Reaction Pathways

In this work, we used the theoretically well-defined free energy diagram (FED) approach proposed by Norskov group. It has been a generally accepted approach for electrochemical studies based on the standard density functional theory (DFT) in combination with the computational standard hydrogen electrode (SHE) model. For the investigation of various catalytic reactions on specific surface structures, we mainly consider the thermodynamic stabilities of the intermediates as the main descriptor, which determines the catalytic performance [S88, S89].

In the case of oxygen evolution reaction (OER), the four-step four-electron reaction has been generally acceptable, where this process in an alkaline environment can be described as follows.

$$OH^- + * \rightarrow OH^* + e^- \tag{S6}$$

$$OH^* + OH^- \rightarrow O^* + H_2O(l) + e^- \tag{S7}$$

$$O^* + OH^- \rightarrow OOH^* + e^- \tag{S8}$$

$$OOH^* + OH^- \rightarrow * + O_2(g) + H_2O(l) + e^- \tag{S9}$$

where * refer to the active site, and OH*, O*, and OOH* are adsorbed intermediates on the surface of catalyst. Similar to the case of OER, in the case of oxygen reduction reaction (ORR), we consider the overall reaction mechanism for the complete direct four-electron process, where O_2 is reduced to water without forming the hydrogen peroxide intermediate, H_2O_2. Also, this mechanism in an alkaline media is the inversed forms of reactions (S6) to (S9) for OER in the following four-step four-electron reaction,

$$O_2(g) + * \rightarrow O_2^* \tag{S10}$$

$$O_2^* + H_2O(l) + e^- \rightarrow OOH^* + OH^- \tag{S11}$$

$$OOH^* + e^- \rightarrow O^* + OH^- \tag{S12}$$

$$O^* + H_2O(l) + e^- \rightarrow OH^* + OH^- \tag{S13}$$

$$OH^{*} + e^{-} \rightarrow * + OH^{-} \quad (S14)$$

where * represents the active site, and O_2*, OOH*, O* and OH* are adsorbed intermediates on the surface.

S4.2 Derivation of the Free Energy Relations

We calculated the reaction Gibbs free energies of the intermediates of O_2*, OOH*, O* and OH* on the bulk CN, NDCN, and polymer-assisted NDCN to determine the potential-determining step of OER and ORR, considering all possible active sites. For each step, the reaction Gibbs free energy ΔG_{ads} can be expressed by

$$\Delta G_{ads} = \Delta E_{ads} + \Delta ZPE - T\Delta S \quad (S15)$$

where ZPE is the zero-point energy, T is the temperature, and ΔS is the entropy change. Using this equation (S15), we can construct a free energy diagram (FED) considering the following four-step four-electron reaction with equations at standard conditions [S90].

The reaction Gibbs free energy of each electrochemical reaction of OER in alkaline media can be expressed as follows:

$$\Delta G_1 = G_{OH*} + \mu_{e-} - (\mu_{OH-} + G_{*}) \quad (S16)$$

$$\Delta G_2 = G_{O*} + \mu_{H2O(l)} + \mu_{e-} - (G_{OH*} + G_{*}) \quad (S17)$$

$$\Delta G_3 = G_{OOH*} + \mu_{e-} - (G_{O*} + \mu_{OH-}) \quad (S18)$$

$$\Delta G_4 = G_{*} + \mu_{O2} + \mu_{H2O(l)} + \mu_{e-} - (G_{OOH*} + \mu_{OH-}) \quad (S19)$$

These reaction Gibbs free energies can be calculated by using the chemical potentials of hydroxide, electron, liquid water, and oxygen molecule (μ_{OH-}, μ_{e-}, $\mu_{H2O(l)}$ and μ_{O2}) and the free energies of each intermediate (G_{OH*}, G_{O*}, G_{OOH*}) on the surface (*).

S4.3 Free Energy Diagram (FED) and Overpotential (η)

From the calculated $\Delta G^{OER/ORR}$ values, we can deduce a critical parameter of electrocatalytic activity, which is the magnitude of the OER and ORR potential-determining step ($G^{OER/ORR}$) in a four-step four-electron reaction. It is the specific reaction point with the largest ΔG in the OER and ORR elementary reaction steps; i.e., the concluding step to achieve an overall downhill reaction in the free energy diagram (FED) with increasing potential (Figs. S14 and S15):

$$G^{OER/ORR} = \max[\Delta G_1, \Delta G_2, \Delta G_3, \Delta G_4]^0 \quad (S20)$$

After finding the largest ΔG value meaning the bottleneck point of ORR, we can finally get the theoretical overpotential in the alkaline condition in the following equation (S21):

$$\eta^{OER/ORR} = (G^{OER/ORR}/e) - xV, x = 0.402 \text{ V} \quad (S21)$$

Supplementary References

[S1] Y. Liang, H. Wang, J. Zhou, Y. Li, J. Wang et al., Covalent hybrid of spinel manganese-cobalt oxide and graphene as advanced oxygen reduction electrocatalysts. J. Am. Chem. Soc. **134**(7), 3517-3523 (2012). https://doi.org/10.1021/ja210924t

[S2] U. Paulus, T. Schmidt, H. Gasteiger, R. Behm, Oxygen reduction on a high-surface area Pt/Vulcan carbon catalyst: a thin-film rotating ring-disk electrode study. J. Electroanal. Chem. **495**(2), 134-145 (2001). https://doi.org/10.1016/S0022-0728(00)00407-1

[S3] S. Zecevic, J. Wainright, M. Litt, S. Gojkovic, R.J. Savinell, Kinetics of O_2 reduction on a Pt electrode covered with a thin film of solid polymer electrolyte. Electrochem. Soc. **144**, 2973-2982 (1997). https://doi.org/10.1149/1.1837946

[S4] O. Antoine, R.J. Durand, RRDE study of oxygen reduction on Pt nanoparticles inside Nafion®: H_2O_2 production in PEMFC cathode conditions. Appl. Electrochem. **30**, 839-844 (2000). https://doi.org/10.1023/A:1003999818560

[S5] Q. Xiang, J. Yu, M. Jaroniec, Preparation and enhanced visible light photocatalytic H_2 production activity of graphene/C_3N_4 composites. J. Phys. Chem. C **115**(15), 7355-7363 (2011). https://doi.org/10.1021/jp200953k

[S6] Y. Wang, X. Bai, C. Pan, J. He, Y. Zhu, Enhancement of photocatalytic activity of Bi_2WO_6 hybridized with graphite like C_3N_4. J. Mater. Chem. **22**(23), 11568-11573 (2012). https://doi.org/10.1039/C2JM16873A

[S7] J. Ou, Y. Zhang, L. Chen, Q. Zhao, Y. Meng et al., Nitrogen-rich porous carbon derived from biomass as a high performance anode material for lithium ion batteries. J. Mater. Chem. A **3**(12), 6534-6541 (2015). https://doi.org/10.1039/C4TA06614F

[S8] Y. Zhang, X. Fan, J. Jian, D. Yu, Z. Zhang et al., A general polymer-assisted strategy enables unexpected efficient metal-free oxygen-evolution catalysis on pure carbon nanotubes. Energy Environ. Sci. **10**(11), 2312-2317 (2017). https://doi.org/10.1039/C7EE01702B

[S9] Y. Cui, J. Zhang, G. Zhang, J. Huang, P. Liu et al., Synthesis of bulk and nanoporous carbon nitride polymers from ammonium thiocyanate for photocatalytic hydrogen evolution. J. Mater. Chem. **21**(34), 13032-13039 (2011). https://doi.org/10.1039/c1jm11961c

[S10] S. Shinde, C. Lee, J. Jung, N. Wagh, S. Kim et al., Unveiling dual-linkage 3D hexaiminobenzene metal-organic frameworks towards long-lasting advanced reversible Zn-air batteries. Energy Environ. Sci. **12**(2), 727-738 (2019). https://doi.org/10.1039/C8EE02679C

[S11] L. Yang, X. Zhang, L. Yu, J. Hou, Z. Zhou et al., Atomic Fe-N_4/C in flexible carbon fiber membrane as binder-free air cathode for Zn-air batteries with stable cycling over 1000 h. Adv. Mater. **34**(5), 2105410 (2021). https://doi.org/10.1002/adma.202105410

[S12] S. Shinde, J. Jung, N. Wagh, C. Lee, S. Kim et al., Ampere-hour-scale zinc-air pouch cells. Nat. Energy **6**, 592-604 (2021). https://doi.org/10.1038/s41560-021-00807-8

[S13] N. Wagh, D. Kim, S. Kim, S. Shinde, J. Lee, Heuristic iron-cobalt-mediated robust pH-universal oxygen bifunctional lusters for reversible aqueous and flexible solid-state Zn-air cells. ACS Nano **15**(9), 14683-14696 (2021). https://doi.org/10.1021/acsnano.1c04471

[S14] Y. Dai, J. Yu, J. Wang, Z. Shao, D. Guan et al., Bridging the charge accumulation and high reaction order for high-rate oxygen evolution and long stable Zn-air batteries. Adv. Funct. Mater. **32**(24), 2111989 (2022). https://doi.org/10.1002/adfm.202111989

[S15] H. Jiang, J. Xia, L. Jiao, X. Meng, P. Wang et al., Ni single atoms anchored on N-doped carbon nanosheets as bifunctional electrocatalysts for Urea-assisted

rechargeable Zn-air batteries. Appl. Catal. B Environ. **310**, 121352 (2022). https://doi.org/10.1016/j.apcatb.2022.121352

[S16] L. Song, T. Zheng, L. Zheng, B. Lu, H. Chen et al., Cobalt-doped basic iron phosphate as bifunctional electrocatalyst for long-life and high-power-density rechargeable zinc-air batteries. Appl. Catal. B Environ. **300**, 120712 (2022). https://doi.org/10.1016/j.apcatb.2021.120712

[S17] L. Zhang, Y. Zhu, Z. Nie, Z. Li, Y. Ye et al., Co/MoC nanoparticles embedded in carbon nanoboxes as robust trifunctional electrocatalysts for a Zn−air battery and water electrocatalysis. ACS Nano **15**(8), 13399−13414 (2021). https://doi.org/10.1021/acsnano.1c03766

[S18] Z. Wu, H. Wang, P. Xiong, G. Li, T. Qiu et al., Molecularly thin nitride sheets stabilized by titanium carbide as efficient bifunctional electrocatalysts for fiber-shaped rechargeable zinc-air batteries. Nano Lett. **20**, 2892-2898 (2020). https://doi.org/10.1021/acs.nanolett.0c00717

[S19] K. Tang, C. Yuan, Y. Xiong, H. Hu, M. Wu, Inverse-opal-structured hybrids of N, S-codoped-carbon-confined Co_9S_8 nanoparticles as bifunctional oxygen electrocatalyst for on-chip all-solid-state rechargeable Zn-air batteries. Appl. Catal. B Environ. **260**, 118209 (2020). https://doi.org/10.1016/j.apcatb.2019.118209

[S20] J. Zhang, Z. Zhao, Z. Xia, L. Dai, A metal-free bifunctional electrocatalyst for oxygen reduction and oxygen evolution reactions. Nat. Nanotechnol. **10**, 444-452 (2015). https://doi.org/10.1038/nnano.2015.48

[S21] J. Yin, Y. Li, F. Lv, Q. Fan, Y. Zhao et al., NiO/CoN porous nanowires as efficient bifunctional catalysts for Zn-air batteries. ACS Nano **11**(2), 2275−2283 (2017). https://doi.org/10.1021/acsnano.7b00417

[S22] S. Shinde, C. Lee, A. Sami, D. Kim, S. Lee et al., Scalable 3-D carbon nitride sponge as an efficient metal-free bifunctional oxygen electrocatalyst for rechargeable Zn-air batteries. ACS Nano **11**(1), 347−357 (2017). https://doi.org/10.1021/acsnano.6b05914

[S23] F. Meng, H. Zhong, D. Bao, J. Yan, X. Zhang, In situ coupling of strung Co_4N and intertwined N-C fibers toward free-standing bifunctional cathode for robust, efficient, and flexible Zn-Air batteries. J. Am. Chem. Soc. **138**(32), 10226-10231 (2016). https://doi.org/10.1021/jacs.6b05046

[S24] X. Wang, Y. Li, T. Jin, J. Meng, L. Jiao et al., Electrospun thin-walled $CuCo_2O_4$@C nanotubes as bifunctional oxygen electrocatalysts for rechargeable Zn-air batteries. Nano Lett. **17**(12), 7989−7994 (2017). https://doi.org/10.1021/acs.nanolett.7b04502

[S25] Z. Pei, H. Li, Y. Huang, Q. Xue, Y. Huang et al., Texturing in situ: N, S-enriched hierarchically porous carbon as a highly active reversible oxygen electrocatalyst. Energy Environ. Sci. **10**(3), 742-749 (2017). https://doi.org/10.1039/C6EE03265F

[S26] M. Zhang, Q. Dai, H. Zheng, M. Chen, L. Dai, Novel MOF-derived Co@N-C bifunctional catalysts for highly efficient Zn-air batteries and water splitting. Adv. Mater. **30**(10), 1705431 (2018). https://doi.org/10.1002/adma.201705431

[S27] Y. Guo, P Yuan, J. Zhang, Y. Hu, I. Amiinu et al., Carbon nanosheets containing discrete Co-N_x-B_y-C active sites for efficient oxygen electrocatalysis and rechargeable Zn-air batteries. ACS Nano **12**(2), 1894−1901 (2018). https://doi.org/10.1021/acsnano.7b08721

[S28] S. Shinde, C. Lee, J. Yu, D. Kim, S. Lee et al., Hierarchically designed 3D holey C_2N aerogels as bifunctional oxygen electrodes for flexible and rechargeable Zn-air batteries. ACS Nano **12**(1), 596-608 (2018). https://doi.org/10.1021/acsnano.7b07473

[S29] L. Ma, S. Chen, Z. Pei, Y. Huang, G. Liang et al., Single-site active iron-based bifunctional oxygen catalyst for a compressible and rechargeable zinc-air battery. ACS Nano **12**(2), 1949−1958 (2018). https://doi.org/10.1021/acsnano.7b09064

[S30] V. Kashyap, S. Kurungot, Zirconium-substituted cobalt ferrite nanoparticle supported N-doped reduced graphene oxide as an efficient bifunctional electrocatalyst for rechargeable Zn-air battery. ACS Catal. **8**(4), 3715−3726 (2018). https://doi.org/10.1021/acscatal.7b03823

[S31] Q. Wang, Y. Ji, Y. Lei, Y. Wang, Y. Wang et al., Pyridinic-N-dominated doped defective graphene as a superior oxygen electrocatalyst for ultrahigh-energy-density Zn-air batteries. ACS Energy Lett. **3**(5), 1183−1191 (2018). https://doi.org/10.1021/acsenergylett.8b00303

[S32] S. Peng, X. Han, L. Li, S. Chou, D. Ji et al., Electronic and defective engineering of electrospun $CaMnO_3$ nanotubes for enhanced oxygen electrocatalysis in rechargeable zinc-air batteries. Adv. Energy Mater. **8**(22), 1800612 (2018). https://doi.org/10.1002/aenm.201800612

[S33] Y. Jiang, Y. Deng, J. Fu, D. Lee, R. Liang et al., Interpenetrating triphase cobalt-based nanocomposites as efficient bifunctional oxygen electrocatalysts for long-lasting rechargeable Zn-air batteries. Adv. Energy Mater. **8**(15), 1702900 (2018). https://doi.org/10.1002/aenm.201702900

[S34] C. Hang, J. Zhang, J. Zhu, W. Li, Z. Kou et al., In situ exfoliating and generating active sites on graphene nanosheets strongly coupled with carbon fiber toward self-standing bifunctional cathode for rechargeable Zn-air batteries. Adv. Energy Mater. **8**(16), 1703539 (2018). https://doi.org/10.1002/aenm.201703539

[S35] H. Yang, J. Miao, S. Hung, J. Chen, H. Tao et al., Identification of catalytic sites for oxygen reduction and oxygen evolution in N-doped graphene materials: development of highly efficient metal-free bifunctional electrocatalyst. Sci. Adv. **2**(4), e1501122 (2016). https://doi.org/10.1126/sciadv.1501122

[S36] Q. Liu, Y. Wang, L. Dai, J. Yao, Scalable fabrication of nanoporous carbon fiber films as bifunctional catalytic electrodes for flexible Zn-air batteries. Adv. Mater. **28**(15), 3000-3006 (2016). https://doi.org/10.1002/adma.201506112

[S37] X. Han, X. Wu, C. Zhong, Y. Deng, N. Zhao et al., $NiCo_2S_4$ nanocrystals anchored on nitrogen-doped carbon nanotubes as a highly efficient bifunctional electrocatalyst for rechargeable zinc-air batteries. Nano Energy **31**, 541-550 (2017). https://doi.org/10.1016/j.nanoen.2016.12.008

[S38] C. Guan, A. Sumboja, H. Wu, W. Ren, X. Liu et al., Hollow Co_3O_4 nanosphere embedded in carbon arrays for stable and flexible solid-state zinc-air batteries. Adv. Mater. **29**(44), 1704117 (2017). https://doi.org/10.1002/adma.201704117

[S39] P. Liu, D. Gao, W. Xiao, L. Ma, K. Sun et al., Self-powered water-splitting devices by core-shell NiFe@N-graphite-based Zn-air batteries. Adv. Funct. Mater. **28**(14), 1706928 (2018). https://doi.org/10.1002/adfm.201706928

[S40] S. Shinde, J. Yu, J. Song, Y. Nam, D. Kim et al., Highly active and durable carbon nitride fibers as metal-free bifunctional oxygen electrodes for flexible Zn-air batteries. Nanoscale Horiz. **2**(6), 333-341 (2017). https://doi.org/10.1039/C7NH00058H

[S41] P. Chen, T. Zhou, L. Xing, K. Xu, Y. Tong et al., Atomically dispersed iron-nitrogen species as electrocatalysts for bifunctional oxygen evolution and reduction reactions. Angew. Chem. Int. Ed. **56**(2), 610-614 (2017). https://doi.org/10.1002/anie.201610119

[S42] G. Li, X. Wang, J. Fu, J. Li, M. Park et al., Pomegranate-inspired design of highly active and durable bifunctional electrocatalysts for rechargeable metal-air batteries. Angew. Chem. Int. Ed. **55**(16), 4977-4982 (2016). https://doi.org/10.1002/anie.201600750

[S43] T. Ma, J. Ran, S. Dai, M. Jaroniec, S. Qiao, Phosphorus-doped graphitic carbon nitrides grown in situ on carbon-fiber paper: flexible and reversible oxygen electrodes. Angew. Chem. Int. Ed. **54**(15), 4646-4650 (2015). https://doi.org/10.1002/anie.201411125

[S44] C. Su, H. Cheng, W. Li, Z. Liu, N. Li et al., Atomic modulation of FeCo-nitrogen-carbon bifunctional oxygen electrodes for rechargeable and flexible all-solid-state zinc-air battery. Adv. Energy Mater. **7**(13), 1602420 (2017). https://doi.org/10.1002/aenm.201602420

[S45] S.Mao, Z. Wen, T. Huang, Y. Hou, J. Chen, High-performance bi-functional electrocatalysts of 3D crumpled graphene-cobalt oxide nanohybrids for oxygen reduction and evolution reactions. Energy Environ. Sci. **7**(2), 609-616 (2014). https://doi.org/10.1039/C3EE42696C

[S46] R. Krishnapriya, S. Praneetha, A.M. Rabel et al. Energy efficient, one-step microwave-solvothermal synthesis of highly electro-catalytic thiospinel $NiCo_2S_4$/graphene nanohybrid as a novel sustainable counter electrode material for Pt-free dye-sensitized solar cells. J. Mater. Chem. C **5**(12), 3146-3155, 2017. https://doi.org/10.1039/C6TC04619C

[S47] G. Wang, J. Chang, S. Koul, A. Kushima, Y. Yang, CO_2 bubble-assisted Pt exposure in PtFeNi porous film for high-performance zinc-air battery. J. Am. Chem. Soc. **143**(30), 11595−11601 (2021). https://doi.org/10.1021/jacs.1c04339

[S48] D. Lyu, S. Yao, A. Ali, Z. Tian, P. Tsiakaras et al., N, S codoped carbon matrix-encapsulated Co_9S_8 nanoparticles as a highly efficient and durable bifunctional oxygen redox electrocatalyst for rechargeable Zn-air batteries. Adv. Energy Mater. **11**(28), 2101249 (2021). https://doi.org/10.1002/aenm.202101249

[S49] H. Zhang, M. Zhao, H. Liu, S. Shi, Z. Wang et al., Ultrastable FeCo bifunctional electrocatalyst on Se-doped CNTs for liquid and flexible all-solid-state rechargeable Zn-air batteries. Nano Lett. **21**(5), 2255−2264 (2021). https://doi.org/10.1021/acs.nanolett.1c00077

[S50] K. Cui, Q. Wang, Z. Bian, G. Wang, Y. Xu, Supramolecular modulation of molecular conformation of metal porphyrins toward remarkably enhanced multipurpose electrocatalysis and ultrahigh-performance zinc-air batteries. Adv. Energy Mater. **11**(46), 2102062 (2021). https://doi.org/10.1002/aenm.202102062

[S51] W. Liu, J. Zhang, Z. Bai, G. Jiang, M. Li et al., Controllable urchin-like $NiCo_2S_4$ microsphere synergized with sulfur-doped graphene as bifunctional catalyst for superior rechargeable Zn-air battery. Adv. Funct. Mater. **28**(11), 1706675 (2018). https://doi.org/10.1002/adfm.201706675

[S52] B. Li, S. Zhang, B. Wang, Z. Xia, C. Tang et al., A porphyrin covalent organic framework cathode for flexible Zn-air batteries. Energy Environ. Sci. **11**(7), 1723-1729 (2018). https://doi.org/10.1039/C8EE00977E

[S53] T. Yang, S. Venkatesan, C. Lien, J. Chang, J. Zen, Nafion/lead oxide–manganese oxide combined catalyst for use as a highly efficient alkaline air electrode in zinc–air battery. Electrochim. Acta **56**(17), 6205-6210 (2011). https://doi.org/10.1016/j.electacta.2011.03.094

[S54] Y. Liu, S. Chen, X. Quan, H. Yu, H. Zhao et al., Boron and nitrogen codoped nanodiamond as an efficient metal-free catalyst for oxygen reduction reaction. J. Phys. Chem. C **117**(29), 14992-14998 (2013). https://doi.org/10.1021/jp4044094

[S55] M. Prabu, K. Ketpang, S. Shanmugam, Hierarchical nanostructured $NiCo_2O_4$ as an efficient bifunctional non-precious metal catalyst for rechargeable zinc-air batteries. Nanoscale **6**(6), 3173-3181 (2014). https://doi.org/10.1039/c3nr05835b

[S56] R. Cao, R. Thapa, H. Kim, X. Xu, M. Kim et al., Promotion of oxygen reduction by a bio-inspired tethered iron phthalocyanine carbon nanotube-based catalyst. Nat. Commun. **4**, 2076 (2013). https://doi.org/10.1038/ncomms3076

[S57] Y. Li, M. Gong, Y. Liang, J. Feng, J. Kim et al., Advanced zinc-air batteries based on high-performance hybrid electrocatalysts. Nat. Commun. **4**, 1805 (2013). https://doi.org/10.1038/ncomms2812

[S58] V. Dhavale, S. Kurungot, Cu-Pt nanocage with 3-D electrocatalytic surface as an efficient oxygen reduction electrocatalyst for a primary Zn-air battery. ACS Catal. **5**(3), 1445-1452 (2015). https://doi.org/10.1021/cs501571e

[S59] J. Lee, G. Park, H. Lee, S. Kim, R. Cao et al., Ketjenblack carbon supported amorphous manganese oxides nanowires as highly efficient electrocatalyst for oxygen reduction reaction in alkaline solutions. Nano Lett. **11**(12), 5362-5366 (2011). https://doi.org/10.1021/nl2029078

[S60] L. An, Y. Li, M. Luo, J. Yin, Y. Zhao et al., Atomic-level coupled interfaces and lattice distortion on CuS/NiS_2 nanocrystals boost oxygen catalysis for flexible Zn-air batteries. Adv. Funct. Mater. **27**(42), 1703779 (2017). https://doi.org/10.1002/adfm.201703779

[S61] L. Li, C. Liu, G. He, D. Fan, A. Manthiram, Hierarchical pore-in-pore and wire-in-wire catalysts for rechargeable Zn- and Li-air batteries with ultra-long cycle life and high cell efficiency. Energy Environ. Sci. **8**(11), 3274-3282 (2015). https://doi.org/10.1039/C5EE02616D

[S62] D. Lee, J. Choi, K. Feng, H. Park, Z. Chen, Advanced extremely durable 3D bifunctional air electrodes for rechargeable zinc-air batteries. Adv. Energy Mater. **4**(6), 1301389 (2014). https://doi.org/10.1002/aenm.201301389

[S63] X. Liu, M. Park, M. Kim, S. Gupta, G. Wu et al., Integrating NiCo alloys with their oxides as efficient bifunctional cathode catalysts for rechargeable zinc-air batteries. Angew. Chem. Int. Ed. **54**(33), 9654-9658 (2015). https://doi.org/10.1002/anie.201503612

[S64] J. Jung, M. Risch, S. Park, M. Kim, G. Nam et al., Optimizing nanoparticle perovskite for bifunctional oxygen electrocatalysis. Energy Environ. Sci. **9**(1), 176-183 (2016). https://doi.org/10.1039/C5EE03124A

[S65] T. Wang, G. Nam, Y. Jin, X. Wang, P. Ren et al., NiFe (Oxy) hydroxides derived from NiFe disulfides as an efficient oxygen evolution catalyst for rechargeable Zn-air batteries: the effect of surface S residues. Adv. Mater. **30**(27), 1800757 (2018). https://doi.org/10.1002/adma.201800757

[S66] G. Fu, Y. Chen, Z. Cui, Y. Li, W. Zhou et al., Novel hydrogel-derived bifunctional oxygen electrocatalyst for rechargeable air cathodes. Nano Lett. **16**(10), 6516−6522 (2016). https://doi.org/10.1021/acs.nanolett.6b03133

[S67] G. Fu, Z. Cui, Y. Chen, Y. Li, Y. Tang et al., Ni_3Fe-N doped carbon sheets as a bifunctional electrocatalyst for air cathodes. Adv. Energy Mater. **7**(1), 1601172 (2017). https://doi.org/10.1002/aenm.201601172

[S68] D. Lee, J. Fu, M. Park, H. Liu, A. Kashkooli et al., Self-assembled NiO/Ni$(OH)_2$ nanoflakes as active material for high-power and high-energy hybrid rechargeable battery. Nano Lett. **16**(3), 1794-1802 (2016). https://doi.org/10.1021/acs.nanolett.5b04788

[S69] J. Wang, H. Wu, D. Gao, S. Miao, G. Wang et al., Self-assembled NiO/Ni$(OH)_2$ nanoflakes as active material for high-power and high-energy hybrid rechargeable battery. Nano Energy **13**, 387-396 (2015). https://doi.org/10.1021/acs.nanolett.5b04788

[S70] X. Zhuang, D. Gehrig, N. Forler, H. Liang, M. Wagner et al., Conjugated microporous polymers with dimensionality-controlled heterostructures for green energy devices. Adv. Mater. **27**(25), 3789-3796 (2015). https://doi.org/10.1002/adma.201501786

[S71] C. Li, X. Han, F. Cheng, Y. Hu, C. Chen et al., Phase and composition controllable synthesis of cobalt manganese spinel nanoparticles towards efficient oxygen electrocatalysis. Nat. Commun. **6**, 7345 (2015). https://doi.org/10.1038/ncomms8345

[S72] Z. Chen, A. Yu, D. Higgins, H. Li, H. Wang et al., Highly active and durable core-corona structured bifunctional catalyst for rechargeable metal-air battery application. Nano Lett. **12**(4), 1946-1952 (2012). https://doi.org/10.1021/nl2044327

[S73] T. You, C. Hu, Designing binary Ru-Sn oxides with optimized performances for the air electrode of rechargeable zinc-air batteries. ACS Appl. Mater. Interfaces **10**(12), 10064−10075 (2018). https://doi.org/10.1021/acsami.7b18948

[S74] P. Li, C. Hu, T. You, P. Chen, Development and characterization of bi-functional air electrodes for rechargeable zinc-air batteries: effects of carbons. Carbon **111**, 813-821 (2017). https://doi.org/10.1016/j.carbon.2016.10.057

[S75] M. Zhao, H. Liu, H. Zhang, W. Chen, H. Sun et al., A pH-universal ORR catalyst with single-atom iron sites derived from a double-layer MOF for superior flexible quasi-solid-state rechargeable Zn-air batteries. Energy Environ. Sci. **14**(12), 6455-6463 (2021). https://doi.org/10.1039/D1EE01602D

[S76] N. Wagh, S. Shinde, C. Lee, J. Jung, D. Kim et al., Densely colonized isolated Cu-N single sites for efficient bifunctional electrocatalysts and rechargeable advanced Zn-air batteries. Appl. Catal. B Environ. **268**, 118746 (2020). https://doi.org/10.1016/j.apcatb.2020.118746

[S77] J. Fu, F. Hassan, J. Li, D. Lee, A. Ghannoum et al., Flexible rechargeable zinc-air batteries through morphological emulation of human hair array. Adv. Mater. **28**(30), 6421-6428 (2016). https://doi.org/10.1002/adma.201600762

[S78] A. Sumboja, M. Lübke, Y. Wang, T. An, Y. Zong et al., All-solid-state, foldable, and rechargeable Zn-air batteries based on manganese oxide grown on graphene-coated carbon cloth air cathode. Adv. Energy Mater. **7**(20), 1700927 (2017). https://doi.org/10.1002/aenm.201700927

[S79] J. Fu, J. Zhang, X. Song, H. Zarrin, X. Tian et al., A flexible solid-state electrolyte for wide-scale integration of rechargeable zinc-air batterie. Energy Environ. Sci. **9**(2), 663-670 (2016). https://doi.org/10.1039/C5EE03404C

[S80] J. Fu, D. Lee, F. Hassan, L. Yang, Z. Bai et al., Flexible high-energy polymer-electrolyte-based rechargeable zinc-air batteries. Adv. Mater. **27**(37), 5617-5622 (2015). https://doi.org/10.1002/adma.201502853

[S81] G. Kresse, J. Hafner, Ab initio molecular dynamics for open-shell transition metals. Phys. Rev. B **48**, 13115 (1995). https://doi.org/10.1103/PhysRevB.48.13115

[S82] G. Kresse, J. Hafner, Ab initio molecular-dynamics simulation of the liquid-metal-amorphous-semiconductor transition in germanium. Phys. Rev. B **49**, 14251-14269 (1993). https://doi.org/10.1103/PhysRevB.49.14251

[S83] G. Kresse, J. Furthmuller, Efficiency of ab-initio total energy calculations for metals and semiconductors using a plane-wave basis set. Comp. Mater. Sci. **6**(1), 15-50 (1996). https://doi.org/10.1016/0927-0256(96)00008-0

[S84] G. Kresse, J. Furthmuller, Efficient iterative schemes for ab initio total-energy calculations using a plane-wave basis set. Phys. Rev. B **54**, 11169-11186 (1996). https://doi.org/10.1103/PhysRevB.54.11169

[S85] J. Wellendorff, K. Lundgaard, A. Mogelhoj, V. Petzold, D. Landis et al., Density functionals for surface science: exchange-correlation model development with Bayesian error estimation. Phys. Rev. B **85**, 235149 (2012). https://doi.org/10.1103/PhysRevB.85.235149

[S86] G. Kresse, D. Joubert, From ultrasoft pseudopotentials to the projector augmented-wave method. Phys. Rev. B **59**, 1758-1775 (1999). https://doi.org/10.1103/PhysRevB.59.1758

[S87] P.E. Blochl, From ultrasoft pseudopotentials to the projector augmented-wave method. Phys. Rev. B **50**, 17953-17979 (1994). https://doi.org/10.1103/PhysRevB.59.1758

[S88] J. Rossmeisl, Z.W. Qu, H. Zhu, G.J. Kroes, J.K. Norskov, Electrolysis of water on oxide surfaces. J. Electroanal. Chem. **607**, 83 (2007). https://doi.org/10.1016/j.jelechem.2006.11.008

[S89] J.K. Norskov, J. Rosmeisl, A. Logadottir, L. Lindqvist, Origin of the overpotential for oxygen reduction at a fuel-cell cathode. J. Phys. Chem. B **108**, 17886 (2004). https://doi.org/10.1021/jp047349j

[S90] I. Man, H. Su, F. Calle-Vallejo, H. Hansen, J. Martinez et al., Universality in oxygen evolution electrocatalysis on oxide surfaces. ChemCatChem **3**(7), 1159-1165 (2011). https://doi.org/10.1002/cctc.201000397
